# Antioxidant Capacity, Anticancer Ability and Flavonoids Composition of 35 Citrus (*Citrus reticulata* Blanco) Varieties

**DOI:** 10.3390/molecules22071114

**Published:** 2017-07-05

**Authors:** Yue Wang, Jing Qian, Jinping Cao, Dengliang Wang, Chunrong Liu, Rongxi Yang, Xian Li, Chongde Sun

**Affiliations:** 1 Laboratory of Fruit Quality Biology/The State Agriculture Ministry Laboratory of Horticultural Plant Growth, Development and Quality Improvement, Zhejiang University, Zijingang Campus, Hangzhou 310058, China; fruit@zju.edu.cn (Y.W.); qj0621@163.com (J.Q.); caojinpingabc@126.com (J.C.); xianli@zju.edu.cn (X.L.); 2 Horticulture Research Institute, Taizhou Academy of Agricultural Sciences, Linhai 317000, China; 3 Citrus Research Institute, Quzhou Academy of Agricultural Sciences, Quzhou 324000, China; dengliangwang001@163.com (D.W.); qzlcr @aliyun.com (C.L.); 4 Forestry Special Production Technology Promotion Center, Xiangshan Bureau of Agriculture and Forestry, Ningbo 315700, China; mrjq@163.com

**Keywords:** *Citrus reticulata*, phenolics contents, flavonoids composition, antioxidant capacities, anticancer abilities

## Abstract

Citrus (*Citrus reticulate* Blanco) is one of the most commonly consumed and widely distributed fruit in the world, which is possessing extensive bioactivities. Present study aimed to fully understand the flavonoids compositions, antioxidant capacities and in vitro anticancer abilities of different citrus resources. Citrus fruits of 35 varieties belonging to 5 types (pummelos, oranges, tangerines, mandarins and hybrids) were collected. Combining li quid chromatography combined with electrospray ionization mass spectrometry (LC-ESI-MS/MS) and ultra-performance liquid chromatography combined with diode array detector (UPLC-DAD), a total of 39 flavonoid compounds were identified, including 4 flavones, 9 flavanones and 26 polymethoxylated flavonoids (PMFs). Each citrus fruit was examined and compared by 4 parts, flavedo, albedo, segment membrane and juice sacs. The juice sacs had the lowest total phenolics, following by the segment membrane. Four antioxidant traits including 2,2-diphenyl-1-picrylhydrazyl (DPPH) radical scavenging activity, ferric reducing antioxidant power (FRAP), oxygen radical absorbance capacity (ORAC) and cupric reducing antioxidant capacity (CUPRAC) were applied for the antioxidant capacities evaluation. Three gastric cancer cell lines, SGC-7901, BGC-823 and AGS were applied for the cytotoxicity evaluation. According to the results of correlation analysis, phenolics compounds might be the main contributor to the antioxidant activity of citrus extracts, while PMFs existing only in the flavedo might be closely related to the gastric cancer cell line cytotoxicity of citrus extracts. The results of present study might provide a theoretical guidance for the utilization of citrus resources.

## 1. Introduction

Citrus (*Citrus reticulate* Blanco) is a tropical or subtropical fruit widely distributed around the world. As one of the most consumed fruits it also has great economic importance. Besides its value as a delicious fruit, its nutritional values are also important. Previous studies have reported a variety of bioactivities of citrus fruit, like antioxidant [1], anticancer [2,3], anti-inflammation [4], anti-fat [5] and anti-diabetes properties [6,7]. Many of the bioactivities are attributed to the phenolics and flavonoids that are abundant in citrus fruit [5,8,9].

Citrus can be classified in several types, including mandarins, tangerines, oranges, pummelos, hybrids, lemons, limes, etc. [10]. Zhejiang Province, an important citrus production region in China, is rich in citrus germplasm resources. Several distinctive local characteristic citrus varieties had been found in this region, such as Ougan (OG), Yuhuanyou (YHY), Mantouhong (MTH), Huyou (HY) and Ponkan (PG). Thus, the citrus varieties in this region might provide some very distinctive resources for the bioactivity study of citrus fruits. 

The present study aimed to carry out a comprehensive investigation on the flavonoid composition and distribution, antioxidant capacity and gastric tumor cytotoxicity of citrus fruits from Zhejiang Province. A total of 35 varieties belonging to five types of citrus fruits were collected for the study. Ultra-performance liquid chromatography (UPLC) and lipid chromatography combined with electrospray ionization mass spectrometry (LC-ESI-MS/MS) were used for identification and quantification of flavonoid compounds. 2,2-Diphenyl-1-picrylhydrazyl (DPPH) radical scavenging activity, ferric reducing antioxidant power (FRAP), oxygen radical absorbance capacity (ORAC) and cupric reducing antioxidant capacity (CUPRAC), four commonly used antioxidant tests, were applied for the antioxidant capacity evaluation. Gastric tumor cell lines were applied for the cytotoxicity evaluation of citrus flavonoid-rich extracts.

## 2. Results

### 2.1. Fruit Basic Quality Index of Different Citrus Varieties

As shown in Table 1, five types (pummelos, oranges, tangerines, mandarins and hybrids) of 35 varieties of citrus fruits were collected and their fruit basic qualities were tested. Significant differences were found in fruit color, weight, edible rate and total soluble solids (TSS) among the 35 tested citrus varieties. MTH showed the highest Citrus Color Index (CCI) value of 16.75, while Qingougan (QOG) showed the lowest CCI at −6.97. Pummelos showed higher weight than the other types of citrus fruits, among which YHY and Mabuwendan (MBWD) had the highest weights at 1754.10 g and 1676.14 g, respectively. Kouzhijin22 (KZJ22) had the lowest weight of 67.97 g. Hongmeiren (HMR) had the highest edible rate of 85.31%, while Zaoxiangyou (ZXY) only had an edible rate of 56.12%. TSS varied from 8.42 to 16.08 °Brix. KZJ22 had the highest TSS value of 16.08 °Brix, followed by Aiyuan31 (AY31), Rinan1 (RN1), Gongchuan (GOC), Mixiagan (MXG), Aiyuan27 (AY27) and Yuanxiaochun (YXC), while QOG and Wuheougan (WHOG) showed the lowest TSS. Correlation analysis showed that CCI and TSS had significant relationships with each other (*r* = 0.690, *p* < 0.01), indicating that citrus varieties with better color look may have better taste.

### 2.2. Total Phenolics and Antioxidant Capacities

Each citrus fruit was divided into four parts from outside to inside: flavedo, albedo, segment membrane and juice sacs. Ultrasonic assistant extraction was used to improve the extraction efficiency according to previous studies [11,12]. As shown in Table 2, Table 3, Table 4 and Table 5, the total phenolics contents varied between the varieties and tissues of citrus fruits.

Among the four citrus fruit parts, the juice sacs had the lowest total phenolics contents (4.18 mg gallic acid equivalents (GAE)/g dry weight of extracts (DW) in CX to 6.39 mg GAE/g DW in Newhall (NH)), followed by the segment membrane (6.27 mg GAE/g DW in CX to 12.56 mg GAE/g DW in GAC) in all the 35 varieties. The total phenolics contents in the flavedo ranged from 11.52 mg GAE/g DW in ZXY to 27.55 mg GAE/g DW in AY30. While the total phenolics contents in the albedo ranged from 10.35 mg GAE/g DW in CX to 27.15 mg GAE/g DW in GAC. For the flavedo and albedo part, 12 citrus varieties had higher total phenolics contents in flavedo than in albedo, including six pummelos (HY, YHY, MBWD, Zaoxiangyou (ZXY), Putaoyou (PTY), Sijiyou (SJY)), three hybrids (AY27, GOC, MXG) and three tangerines (OG, QOG, WHOG). Zhang et al. [13] had previously tested the total phenolics of 14 wild mandarin genotypes and two cultivars, which ranged from 29.38 to 51.14 mg GAE/g DW. The total phenolics contents of the sum of flavedo and albedo (22.02 to 47.39 mg GAE/g DW) were close to the results of Zhang et al. [13]. 

The DPPH radical scavenging activity, FRAP, ORAC and CUPRAC assays were used to measure the antioxidant capacities of the four citrus parts. The mechanisms of these four antioxidant tests can be divided into two types: the DPPH, FRAP and CUPRAC tests are mainly electron transfer type antioxidant methods [14,15,16,17] while ORAC is a hydrogen supply ability type antioxidant method [18,19]. The results showed significant differences among different varieties for the four fruit parts with each measurement method.

DPPH antioxidant tests are commonly used in determining the primary antioxidant capacities. The DPPH radical scavenging mechanisms include two types: the electron transfer type when components dissolve in polar solutions and the hydrogen supply ability type in nonpolar solutions [14,20]. In this study, the DPPH radical scavenging abilities were mainly based on the electron transfer ability of the antioxidant components since the citrus extracts were dissolved in water. DPPH values ranged from 5.48 to 20.73 mg Trolox equivalent antioxidant capacities (TEAC)/g DW in flavedo and from 4.37 to 17.43 mg TEAC/g DW in albedo. AY30 in flavedo and PG in albedo ranked the first, respectively, while CX showed the lowest value in both parts. In the segment membrane, DPPH value varied from 2.72 (TCH) to 7.34 mg TEAC/g DW (HMR). In the juice sacs, TC had the highest DPPH value (5.73 mg TEAC/g DW) and KZJ22 had the lowest (1.46 mg TEAC/g DW). 

FRAP is another antioxidant test widely used in the determination of plant antioxidant capacity [15]. FRAP values in flavedo varied from 1.94 to 21.33 mg TEAC/g DW, with the highest and lowest value corresponding to AY30 and CX, respectively, which is similar to the DPPH result. GAC showed the highest FRAP value in both albedo (25.26 mg TEAC/g DW) and segment membrane part (19.83 mg TEAC/g DW) while YHY showed the lowest value in albedo (4.82 mg TEAC/g DW). CX ranked the last in flavedo, segment membrane, juice sacs, which may due to its low total phenolics content. FRAP value of AY27 (13.00 mg TEAC/g DW) ranked the first in juice sacs, which is almost three times higher than CX (4.28 mg TEAC/g DW).

The ORAC method, which was widely used in animal and botany materials, tests the hydrogen atom transfer ability of antioxidant components [18]. The ORAC values were the highest among the four methods, which may due to its unique antioxidant type. TC showed the highest ORAC value in flavedo (468.96 mg TEAC/g DW) and albedo (677.90 mg TEAC/g DW), while NG20 showed the lowest value in flavedo (75.78 mg TEAC/g DW) and AY27 showed the lowest in albedo (55.49 mg TEAC/g DW). In the segment membrane, YHY and YXC showed the highest value (208.86 mg TEAC/g DW) and lowest value (37.26 mg TEAC/g DW), respectively. In juice sacs parts, interestingly, KZJ22 showed the highest ORAC value of 76.53 mg TEAC/g DW, although KZJ22 showed the lowest DPPH value in the same part, suggesting that different antioxidant measurements could show great differences in the antioxidant index.

The CUPRAC assay determines the capacities of tested materials to reduce divalent copper ions to cuprous ions [17]. The CUPRAC value ranking was similar to that of the FRAP value, which may be due to the similar antioxidant mechanisms involved. AY30 showed the highest CUPRAC value in flavedo (54.80 mg TEAC/g DW), OG ranked the first in albedo (46.63 mg TEAC/g DW), while ZXY showed the lowest CUPRAC value in both parts (24.44 mg TEAC/g DW in flavedo and 11.34 mg TEAC/g DW albedo). In segment membrane, HY showed the highest value (6.37 mg TEAC/g DW) and CX showed the lowest value (2.57 mg TEAC/g DW). In juice sacs, HY ranked the first with 6.54 mg TEAC/g DW and KZJ22 ranked the last with CUPRAC value of 2.30 mg TEAC/g DW.

To comprehensively compare the antioxidant capacities, an overall antioxidant potency composite (APC) index was calculated according to the method described by Seeram et al. [21]. The overall APC index showed obvious variations, ranging from 33.03 (SJY) to 92.01 (AY30) in flavedo, from 24.96 (YHY) to 86.39 (GAC) in albedo, from 40.08 (TCH) to 90.96 (GAC) in segment membrane, and from 50.03 (YXC) to 84.23 (AY27) in juice sacs

### 2.3. Tumor Cytotoxicity

In vitro tumor cytotoxicity of the citrus extracts were measured on three gastric cancer cell lines, i.e., SGC-7901, BGC-823 and AGS. Cell viability assays were performed with a Cell Counting Kit-8 (CCK-8) and the corresponding IC_50_ values were calculated. Among the four parts, only flavedo extracts of each citrus variety showed significant tumor cytotoxicity, with IC_50_ values as shown in Table 6, while the other three parts showed no significant cytotoxicity effects (data not shown). Among the three cancer cell lines, the AGS cell line seem to be generally more sensitive to citrus extracts treatments than other two cell lines, which can be inferred from the lower IC_50_ values. Among the citrus types, pummelo fruits extracts showed the high IC_50_ value (>100 μg/mL) in all three cell lines, suggested that the tumor cytotoxicity of pummelo fruits to the cancer cells tested was weak. QJ (IC_50_ value = 20.36 μg/mL), CR (IC_50_ value = 18.71 μg/mL), NH (IC_50_ value = 15.77 μg/mL) showed the highest antitumor activity to SGC-7901, BGC-823, AGS cell, respectively. The highest IC_50_ value for each cell is more than 15 times higher than the lowest.

### 2.4. Identification and Quantification of Individual Flavonoid Compound

Further identification and quantification of individual flavonoid in citrus fruits were performed by LC-ESI-MS/MS and UPLC-DAD. A total of 39 flavonoid compounds, including four flavones, nine flavanones and 26 PMFs, were identified. The identification was based on comparison of the retention times and the maximum absorption wavelength of standards, as well as the fragment ion information reported in previous studies (Table 7 and Table S6, Figures S1–S5) [22,23,24].

The four flavones included three flavone *C*-glucosides (vicenin-2, apigenin-8-*C*-glusoide and diosmetin-6-*C*-glucoside) and one flavone *O*-glycoside (rhoifolin). Diosmetin-6-*C*-glucoside was only found in the Buzhihuo (BZH), NG20, SJY and MXG, with the highest concentration of 9.05 mg/g DW in the albedo part of BZH. Rhoifolin was only found in pomelo type fruit (Table S1 and S3–S5).

The nine flavanone *O*-glycosides included eriocitrin, neoeriocitrin, narirutin, naringin, hesperidin, neohesperidin, didymin, poncirin and melitidin, in which narirutin, naringin, hesperidin, neohesperidin were the most common flavonoid components in the 35 citrus varieties (Table S1 and 3–S5). Naringin was very abundant in pummelo type fruit. The content of narirutin was generally low in the pomelo type fruit. Melitidin was only found in the flavedo part of MXG, while vicenin-2 was only existed in KZJ22. The content of hesperidin and neohesperidin showed a seesawing like relationship, that is, the varieties with abundant hesperidin tend to have low level of neohesperidin, which may due to the differentiation in synthetic metabolism. 

The PMFs were only found in flavedo, including one trihydroxydimethoxyflavone, four trimethoxyflavones, seven tetramethoxyflavones, eight pentamethoxyflavones, five hexa-methoxyflavones and one heptamethoxyflavone (Table S2). Among the 26 PMFs identified, there were 12 monohydroxy PMFs, one dihydroxy PMFs and one trihydroxy PMF. The exact location of the methoxy groups of two monohydroxypentamethoxyflavones, two hexamethoxyflavones and three monohydroxypentamethoxyflavones need further identification and we added a number after their names according to the order they appeared in the UPLC chromatogram. The PMF contents differed among varieties. Trihydroxydimethoxyflavone only existed in HMR (3.78 mg/g DW) and 5,7,3′,4′,5′-pentamethoxyflavone only existed in AY27 (1.22 mg/g DW). Isosinensetin sinensetin, tetramethyl-*O*-isoscutellarein, nobiletin, tetramethyl-*O*-scutellarein, 3,5,6,7,8,3′,4′-heptamethoxy-flavone, tangeretin, 5-hydroxy-6,7,8,3′,4′-pentamethoxyflavone existed in most citrus varieties. Nobiletin was the maximum PMFs in almost all of the varieties except for TCH, WZ, DF, SYXX, SW and MXG.

### 2.5. Correlations between Total Phenolics and Bioactivites

Correlation analyses were performed to investigate the relationship between the phenolics content, antioxidant ability and cytotoxicity on gastric cancer cell (Table 8 and Table 9).

For the 4 antioxidant traits, DPPH, FRAP, CUPRAC showed significant correlation with each other (*p* < 0.01), indicating that these 3 traits determined the same type of antioxidant capacities. However, ORAC method only showed correlation with other 3 traits in flavedo. In other 3 parts of citrus, ORAC traits showed very weak relation with other 3 methods, suggesting that ORAC value reflected a different type of antioxidant ability. Total phenolics contents in all the 4 parts showed significant correlations with all 4 the antioxidant traits and the APC overall index (Table 8 and Table 9), indicating that phenolics compounds were the principle contributor to antioxidant capacities of citrus. High correlation of total phenolics contents and antioxidant capacities also showed in the peach fruit [25], Chinese bayberry [26], vegetables and grains [27], indicating that this is a common phenomenon in nature.

The cytotoxicity of extracts showed high correlations among 3 cell traits. However, the correlations between antioxidant capacities and anticancer abilities were relatively low, indicating that the in vitro cytotoxicity of citrus extracts may not be caused by its antioxidant ability. In plenty previous anticancer studies, most of the flavanones were reported to be functioned through enhancing the body’s own function which was based on antioxidant capacities of flavanone [28,29,30]. However, the results of present study indicated a different functional mechanism of citrus flavonoids rich extracts. 

**Figure 1:**
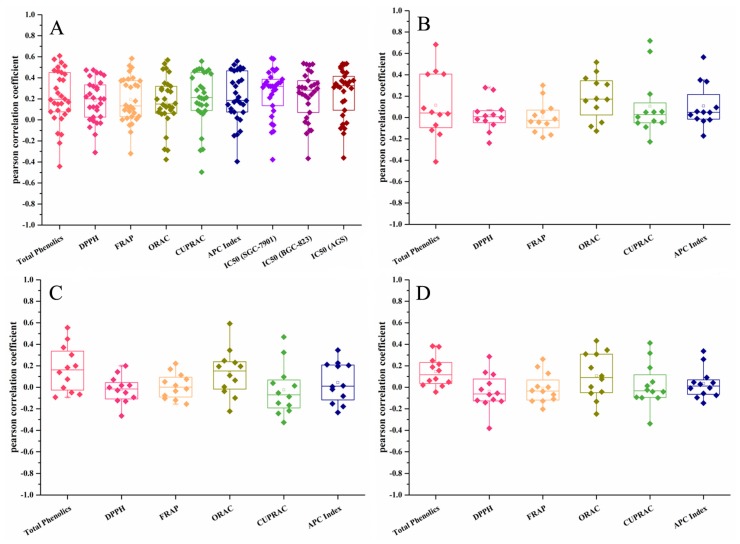
Correlation analysis of bioactive traits and individual flavonoid compounds in flavedo (**A**); albedo (**B**); segment membrane (**C**) and juice sacs (**D**).

For the cytotoxicity of individual compounds, the contents of 11 PMFs showed significant correlations with cytotoxicity of extracts on all three gastric cancer cell lines, in which nobiletin showed the highest correlation coefficient (*r _SGC-7901_* = 0.587, *p* < 0.01; *r _BGC-823_* = 0.530, *p* < 0.01; *r _AGS_* = 0.534, *p* < 0.01) (Figure 1, Tables S7–S10). None of the flavanones or flavones showed significant positive relationships with cytotoxicity and rhoifolin showed a significant negative relationship (*r _SGC-7901_* = −0.378, *p* < 0.05; *r _BGC-823_* = −0.366, *p* < 0.05; *r _AGS_* = −0.361, *p* < 0.05). These results suggested that PMFs might be the main contributors to the cytotoxicity of extracts. Similar results were observed in previous studies. PMFs have shown anti-proliferation abilities to many cancers in vitro [31,32] and in vivo [33,34]. They were suggested to be functioned through induction of cell apoptosis [33,35], cell cycle blocking [36,37] and autophagy, etc. [38,39].

For the antioxidant activity of individual compounds, the didymin showed the positive relationship with APC index and all four antioxidant tests in flavedo. Hesperidin in flavedo (*r* = 0.558, *p* < 0.01), neohesperidin in albedo (*r* = 0.718, *p* < 0.01), poncirin (*r* = 0.618, *p* < 0.01) in albedo showed strong relationship with CUPRAC. Naringin showed significant relationship with ORAC value in segment membrane (*r* = 0.592, *p* < 0.01). These results showed that didymin, hesperidin, neohesperidin, poncirin, naringin might played dominant roles in antioxidant ability of citrus extracts.

## 3. Experimental Section

### 3.1. Materials

Citrus fruits at commercial maturity were harvested from orchards of Zhejiang Province in December 2015 (Table 1), and transported to the laboratory of Zhejiang University, Hangzhou, within 6 h of harvest. Uniform fruit free from blemishes and mechanical injury was selected for the present study. The fruits were separated into four parts, i.e., flavedo, albedo, segment membrane and juice sacs, and immediately frozen in liquid nitrogen. After freeze-drying (FM 25EL-85, VirTis, Gardiner, NY, USA), all samples were ground into a fine powder and stored at −80 °C for further experiments. The SGC-7901, BGC-823, AGS gastric cancer cell lines were obtained from the Department of Surgery, Second Affiliated Hospital, School of Medicine, Zhejiang University.

### 3.2. Chemicals and Reagents

All standards and reagents were of HPLC grade. Narirutin, neoeriocitrin, hesperidin, naringenin, poncirin, naringin, hesperidin, neohesperidin, nobiletin, tangeretin, 2,2-diphenyl-1-picrylhydrazyl (DPPH), 2,4,6-tris(2-pyridyl)-s-triazine (TPTZ), Trolox, Folin-Ciocalteu reagent, 2,2′-azobis(2-methylpropionamidine) dihydrochloride (AAPH), rutin, gallic acid, fluorescein sodium, copper chloride, neocuproine, ammonium acetate, methanol, acetonitrile were purchased from Sigma-Aldrich (St. Louis, MO, USA). Eriocitrin was purchased from Aladdin Industrial Inc. (Shanghai, China). Isosinensetin, and 5-demethylnobiletin were purchased from Biobiopha Co., Ltd. (Kunming, China). Didymin was purchased from J & K Scientific (Shanghai, China). Cell Counting Kit-8 was purchased from Dojindo Molecular Technologies, Inc. (Shanghai, China). Samples for HPLC were filtered through a 0.22 μm membrane before injection. Double-distilled water (ddH_2_O) was used in all experiment. RPMI 1640 medium, fetal bovine serum (FBS), *N*-2-hydroxyethylpiperazine-*N*-2-ethane sulfonic acid (HEPES), trypsin-EDTA were purchased from Gibco (Waltham, MA, USA). Penicillin-streptomycin solution was purchased from Hangzhou Keyi Biotechnology Co., Ltd. (Hangzhou, China). All other solvents and reagents were of analytical grade purchased from Sinopharm Chemical Reagents Co., Ltd. (Shanghai, China).

### 3.3. Fruit Quality Analysis (Color, Fruit Weight, Edible Proportion and Total Soluble Sugar)

Fruit color measurement was carried out using the Citrus Color Index (CCI) according to a previous report [40]. The raw data were adopted as L*, a* and b* with a MiniScan XE Plus Colorimeter (HunterLab, Reston, VA, USA) and the CCI was calculated as CCI = [1000 × a*/(L* × b*)]. Four evenly distributed equatorial sites were measured for each fruit and a mean value was obtained from the measurement of 15 fruits per variety. The edible rate was calculated as the weight percentage of pulp to the whole fruit. TSS of 15 fruits per variety were measured with a portable digital refractometer (Atago PR-101α, Tokyo, Japan) at 25 °C and the data were expressed as °Brix.

### 3.4. Extraction and Determination of Total Phenolics

One gram of citrus fruit ground powder was ultrasonically extracted in 20 mL of 95% ethanol at 25 °C in a material-to-solvent ratio of 1:20 (*w/v*) for three times. The extract was centrifuged at 10,000 ×*g* for 5 min and the supernatants were evaporated under reduced pressure at 35 °C to remove the ethanol. The phenolics were enriched by solid-phase extraction using a Sep-pak C18 cartridge (12 cc, 2 g sorbent, Waters Corp., Milford, MA, USA). The citrus phenolic-rich extracts were used for further analysis.

Total phenolics contents were measured with a Folin-Ciocalteu method according to previous report [41] with slight modification. In brief, 4 mL of ddH_2_O and 0.5 mL appropriately-diluted citrus extracts was placed into a test tube, added with 0.5 mL Folin-Ciocalteu (0.5 mol/L) and incubated for 3 min. Then 1 mL of saturated sodium carbonate was added into the mixture following by incubating the reaction for 2 h in 30 °C water bath. The absorbance of the reaction product were measured at 760 nm using a microplate reader (Synergy H1, Biotek, Winooski, VT, USA). Gallic acid was used as the standard and the results were expressed as mg gallic acid equivalent (GAE)/g DW.

### 3.5. Antioxidant Capacity Assays

The DPPH radical scavenging activity was measured according to our previous publication [42] with some modification. In brief, 2 μL diluted citrus extracts was mixed with 198 μL DPPH solution (60 μM), the mixture was allowed to react for 2 h at room temperature, away from light. Then the absorbance at 515 nm was measured using a microplate reader. Trolox was used as the standard and the results were expressed as mg Trolox equivalent antioxidant capacities (TEAC)/g DW.

The FRAP assay was carried out according to Zhang et al. [43] with modifications. Briefly, the FRAP working solution was prepared by mixing 100 mL acetate buffer (300 mmol/L, pH 3.6), 10 mL TPTZ solution (10 mmol/L in 40 mmol/L HCl) and 10 mL FeCl_3_ (20 mmol/L). 10 μL appropriately diluted citrus extracts and 90 μL of FRAP working solution was mixed in a 96-well plate and incubated for 5 min. Then the absorbance of 593 nm was recorded with a microplate reader. Trolox was used as the standard and the results were expressed as mg Trolox equivalent antioxidant capacities (TEAC)/g DW.

ORAC antioxidant activity was measured according to previous report [44] with some minor modifications. 25 μL appropriately diluted citrus extraction was placed into a black-walled 96-well plate, and mixed with 150 μL sodium fluorescein (40 nmol/L). The mixture was incubated for 10 min at 37 °C. Then 25 μL AAPH (150 mmol/L) was added and the fluorescence detection was performed immediately with a microplate reader (set with excitation wavelength of 485 nm, emission wavelength of 535 nm, time interval of 2 min for the 2 h detection). Phosphate buffer solution (PBS) was used as a blank control and the final fluorescence measurements were expressed relative to the initial reading (f_n_). Results were calculated based on the differences in areas under the sodium fluorescein decay curve (AUC) between the blank and samples. Trolox was used as standard and the results were expressed as mg Trolox equivalent antioxidant capacities (TEAC)/g DW. The AUC was calculated as:

AUC = (f_0_ + f_1_ + f_2_ + … + f_n_)/f_0_

The CUPARC assay was carried out according to previous report [16] with some modifications. Reaction system consisted of 20 μL appropriately diluted citrus extraction, 50 μL copper chloride (10 mmol/L), 50 μL neocuproine (7 mmol/L), 50 μL ammonium acetate (pH 7) and 50 μL ddH_2_O was added into a 96-well plate in order. After 30 min of incubation away from light in room temperature, absorbance of 450 nm was measured in a microplate reader. Trolox was used as standard and the results were expressed as mg Trolox equivalent antioxidant capacities (TEAC)/g DW. 

An overall APC index was applied to comprehensively evaluated the antioxidant traits of the extracts. For each antioxidant trait, antioxidant index score = [(sample score/best score) × 100]. The APC index was calculated as the average of the antioxidant index scores of the referred 4 methods.

### 3.6. Cell Culture and Cell Viability Assay

The human gastric cancer cell lines SGC-7901, BGC-823, AGS were cultured in RPMI 1640 medium containing 10% fetal bovine serum (FBS), 100 U/mL penicillin, 100 μg/mL streptomycin, and 20 mmol/L HEPEs, at 37 °C in an incubator (Thermo Scientific 3111, Thermo Scientific, Waltham, MA, USA) containing 5% CO_2_. Cells were passaged every 48 h using trypsin (0.25%)/EDTA (0.02%) solution. Exponentially growing cells were used for experimentation.

Cell viability assay was performed with cell counting kit-8 (CCK-8) analysis according to the methods described in previous study [45]. Briefly, cells (4000 cells per well for SGC-7901, 8000 cells per well for BGC-823 and 9000 cells per well for AGS) were seeded into 96-well plates. After 24 h incubation, the medium were moved and cells were treated with or without citrus extractions in a total volume of 200 μL each well. After 48 h incubation, the supernatant was removed and washed with PBS for 2 times. The cell viability was measured using CCK-8 kit according to the instruction. Taxol was used as positive control. The inhibition ratio was calculated:

Inhibition ratio = (A_450_ − A_620_)/A_450_. (1)

The IC50 value was calculated by probit analysis method using SPSS 19.0 software (IBM, Armonk, NY, USA).

### 3.7. UPLC-DAD and LC-ESI-MS/MS Analysis of Phonilic Compounds

Individual flavonoid compounds were identified and quantified combining LC-ESI-MS/MS and UPLC-DAD. Flavones and flavanones were detected at 280 nm and polymethoxylated flavonoids were detected at 330 nm. 13 flavanoids, i.e., eriocitrin, neoericitrin, narirutin, naringin, hesperidin, neohesperidin, didmin, poncirin, isosinensetin, sinensitin, nobiletin, tangeretin, 5-hydroxy-6,7,8,3′,4′-pentamethoxyflavone, were quantified with their own standard curves according to the retention time and the chromatographic peak area in the UPLC analysis. Other 26 flavonoids were quantified as equivalents of hesperidin at 280 nm. All tests were run in triplicate and data were expressed as mg/g DW.

The flavonoid compounds were determined with a UPLC system (2695 pump, 2996 diode array detector, Waters Corp.) coupled with an BEH C18 analytical column (ACQUITY UPLC, 2.1 × 150 mm, Waters Corp.). The column was operated at a temperature of 25 °C. The injection of sample was 2 μL and the flow rate was 0.3 mL/min. The compounds were detected between 200 and 500 nm. The mobile phase of UPLC consisted waters (Eluent A) and acetonitrile (Eluent B). the gradient program was as follows: 0–5 min, 20% of B; 5–8 min, 20–34% of B; 8–20 min, 34–60% of B; 20–22 min, 60–100% of B; 22–23 min, 100% of B; 23–24 min, 100–20% of B; 24–25 min, 20% of B.

Mass spectrometric analysis were performed according to our previous publication [25]. Briefly, an Agilent 6460 triple quadrupole mass spectrometer equipped with an ESI source (Agilent Technologies, Santa Clara, CA, USA) was used for mass analysis and the analysis were operated in positive ionization mode. The nebulizer pressure was set to 45 psi and drying gas flow rate was 5 L/min. The flow rate and the temperature of the sheath gas was 11 L/min and 350 °C, respectively. Chromatographic separations were done on an BEH C18 analytical column (ACQUITY UPLC, 2.1 × 150 mm) using an Agilent 1290 Infinity UPLC system (Agilent Technologies). The eluent was split and with a rate of 0.3 mL/min going into the mass detector. The data acquisition and processing were performed at an Agilent Mass Hunter Workstation.

### 3.8. Statistical Analysis

All data were obtained from at least three replications and expressed as the means ± standard deviation. The statistical analyses were carried out using SPSS 19.0 software (IBM, Armonk, NY, USA). Significant differences among the sample were analyzed using one-way ANOVA, followed by Tukey’s test at *p* < 0.05. Pearson correlation coefficients were calculated at *p* < 0.05.

## 4. Conclusions

A total of 39 flavonoids, including four flavones, nine flavanones and 26 PMFs were identified and quantified from 35 varieties of five types of citrus fruit growing in Zhejiang Province of China. Among them, all 39 compounds could be found in the flavedo, three flavones and nine flavanones were found in the albedo, segment membrane and juice sacs, while PMFs were existing only in the flavedo. The flavonoids composition and bioactivity varied depending on the types and tissues of citrus fruit. According to the results of correlation analysis, phenolics were deduced to be the chief contributor for the antioxidant capacity of citrus fruit, in which the individual flavanone compounds including didymin, hesperidin, neohesperidin, poncirin, naringin were the principal contributing components. Phenolics extracts from flavedo showed significant cytotoxicity effects on gastric tumor cell lines, and PMFs were deduced to be the dominant contributors, with the nobiletin as the principle contributing component. The correlation between antioxidant capacities and the cytotoxicity effects was not significant. These results may offer important information for breeding and further utilization of citrus resources.

## Figures and Tables

**Table 1:** Appearance and taste qualities of *Citrus* fruits of 35 cultivars. TSS = total soluble solids.

Cultivar (Abbreviation)	Fruit Type	Color (CCI)	Weight (g) *	Edible Rate (%)	TSS (°Brix) *
Aiyuan27 (AY27)	Hybrids	5.09 ± 0.09	397.97 ± 27.47 ^ef^	84.18	12.85 ± 0.05 ^abcdef^
Aiyuan30 (AY30)	Hybrids	14.10 ± 0.26	114.11 ± 11.15 ^lmno^	80.06	12.51 ± 0.29 ^bcdefg^
Aiyuan31 (AY31)	Hybrids	13.05 ± 0.32	385.06 ± 21.19 ^efg^	84.54	14.79 ± 0.04 ^ab^
Buzhihuo (BZH)	Hybrids	4.69 ± 0.38	216.62 ± 12.05 ^ij^	75.95	10.91 ± 0.50 ^cdefghij^
CaRaCaRa (CR)	Oranges	5.63 ± 1.85	159.36 ± 2.56 ^ijklm^	75.12	11.87 ± 0.60 ^bcdefghi^
Chunxiang (CX)	Hybrids	−4.95 ± 0.44	200.58 ± 9.23 ^ijk^	67.34	9.17 ± 0.28 ^ghij^
Dafen (DF)	Mandarins	3.29 ± 0.33	120.31 ± 12.15 ^klmno^	79.82	10.49 ± 0.28 ^cdefghij^
Gaocheng (GAC)	Hybrids	7.35 ± 0.09	343.87 ± 20.94 ^gh^	74.51	12.44 ± 0.25 ^bcdefgh^
Gongneiyiyugan (GN)	Hybrids	2.76 ± 0.66	241.55 ± 21.74 ^ij^	74.14	10.21 ± 0.56 ^cdefghij^
Gongchuan (GOC)	Mandarins	8.27 ± 0.33	141.94 ± 5.75 ^jklmno^	82.51	13.23 ± 0.45 ^abcd^
Hongmeiren (HMR)	Hybrids	7.59 ± 0.49	137.70 ± 4.03 ^jklmno^	85.31	11.10 ± 0.08 ^cdefghij^
Huyou (HY)	Pummelos	1.43 ± 0.80	571.61 ± 46.24 ^d^	67.97	9.66 ± 0.08 ^efghij^
Kouzhijin22 (KZJ22)	Hybrids	14.68 ± 0.22	67.97 ± 3.80 ^o^	67.59	16.08 ± 0.71 ^a^
Mabuwendan (MBWD)	Pummelos	1.22 ± 0.05	1676.14 ± 81.74 ^a^	59.74	8.93 ± 0.08 ^cdefghij^
Mantouhong (MTH)	Tangerines	16.75 ± 0.63	84.27 ± 3.26 ^no^	79.89	12.53 ± 0.38 ^bcdefg^
Mixiagan (MXG)	Hybrids	1.97 ± 0.08	303.59 ± 18.62 ^gh^	72.13	12.96 ± 0.25 ^abcde^
Nangan20 (NG20)	Mandarins	9.22 ± 0.12	100.61 ± 3.60 ^mno^	74.07	11.76 ± 0.15 ^bcdeghi^
Newhall (NH)	Oranges	8.72 ± 0.38	168.28 ± 5.15 ^ijklm^	71.66	11.96 ± 0.70 ^bcdefghi^
Ougan (OG)	Mandarins	−0.56 ± 1.09	175.42 ± 3.55 ^ijklm^	64.12	9.46 ± 0.22 ^fghij^
Ponkan (PG)	Tangerines	4.89 ± 0.94	140.61 ± 2.34 ^jklmno^	74.68	9.08 ± 0.12 ^hij^
Putaoyou (PTY)	Pummelos	0.24 ± 1.02	442.38 ± 21.67 ^e^	77.49	9.56 ± 0.23 ^fghij^
Qingji (QJ)	Hybrids	6.92 ± 0.20	170.73 ± 8.25 ^ijklm^	73.18	10.55 ± 0.41 ^cdefghij^
Qingougan (QOG)	Mandarins	−6.97 ± 0.44	146.74 ± 3.78 ^ijklmno^	69.33	8.42 ± 0.05 ^j^
Rinan1 (RN1)	Mandarins	8.99 ± 0.08	118.75 ± 5.82 ^klmno^	76.04	13.48 ± 0.14 ^abc^
Shangyexinxi (SYXX)	Mandarins	9.09 ± 0.20	228.56 ± 5.10 ^hi^	80.21	10.34 ± 0.36 ^cdefghij^
Shiwen (SW)	Mandarins	3.12 ± 0.30	74.16 ± 1.31 ^o^	80.46	11.54 ± 0.09 ^bcdefghi^
Sijiyou (SJY)	Pummelos	1.70 ± 0.19	1486.37 ± 21.38 ^b^	65.72	9.68 ± 0.30 ^efghij^
Tiancao (TC)	Hybrids	7.32 ± 0.90	138.75 ± 2.68 ^jklmno^	85.24	9.89 ± 0.42 ^defghij^
Tianchun (TCH)	Hybrids	2.86 ± 0.21	188.85 ± 6.50 ^ijkl^	69.11	11.22 ± 0.18 ^cdefghij^
Weizhang (WZ)	Mandarins	8.39 ± 0.54	128.33 ± 4.86 ^klmno^	73.29	10.05 ± 0.22 ^defghij^
Wuheougan (WHOG)	Mandarins	−1.09 ± 0.98	113.50 ±4.12 ^lmno^	64.25	8.74 ± 0.18 ^ij^
Youliang (YL)	Mandarins	10.45 ± 0.17	138.13 ± 12.12 ^jklmno^	75.32	12.22 ± 0.50 ^bcdefgh^
Yuanxiaochun (YXC)	Hybrids	1.34 ± 0.07	135.94 ± 6.26 ^jklmno^	79.54	12.76 ± 0.42 ^abcdef^
Yuhuanyou (YHY)	Pummelos	0.68 ± 0.08	1754.10 ± 76.73 ^a^	73.10	10.92 ± 0.20 ^cdefghij^
Zaoxiangyou (ZXY)	Pummelos	1.80 ± 0.18	1585.77 ± 76.21 ^b^	56.12	11.38 ± 0.09 ^cdefghij^

* Results were the mean ± SD (*n* = 15) on a fresh weight (FW) (g) and TSS (°Brix) of citrus fruit. Values within each column followed by different superscript letters were significantly different at *p* < 0.05 according to Tukey’s tests.

**Table 2:** Total phenolics and antioxidant properties of citrus fruit flavedo of 35 cultivars.

Cultivars	Total Phenolics	DPPH	FRAP	ORAC	CUPRAC	APC Index *	Rank ^#^
AY27	15.05 ± 0.56 ^no^	11.36 ± 0.71 ^jklm^	9.63 ± 0.44 ^hijk^	153.73 ± 8.78 ^kl^	29.35 ± 1.09 ^pqr^	46.57	26
AY30	27.55 ± 0.29 ^a^	20.73 ± 0.12 ^a^	21.33 ± 1.31 ^a^	319.01 ± 44.63 ^bc^	54.80 ± 2.34 ^a^	92.01	1
AY31	13.73 ± 0.36 ^op^	9.99 ± 0.93 ^mno^	7.12 ± 0.24 ^lmnop^	188.39 ± 15.19 ^hijkl^	32.85 ± 3.32 ^nop^	45.42	28
BZH	19.68 ± 0.58 ^defg^	16.91 ± 0.24 ^bc^	12.36 ± 1.65 ^efg^	221.30 ± 13.70 ^defghijkl^	42.76 ± 0.59 ^defg^	66.18	7
CR	18.90 ± 0.45 ^ghi^	15.34 ± 0.80 ^bcdef^	11.94 ± 0.48 ^efgh^	224.91 ± 26.69 ^defghijk^	42.69 ± 0.59 ^defg^	63.96	10
CX	11.67 ± 0.40 ^q^	5.48 ± 0.56 ^r^	1.94 ± 0.16 ^s^	162.38 ± 18.82 ^jkl^	27.62 ± 0.34 ^qrs^	30.14	35
DF	18.07 ± 0.41 ^ghijk^	10.29 ± 0.83 ^lmn^	7.93 ± 0.30 ^klmno^	206.96 ± 18.53 ^efghijkl^	37.07 ± 0.65 ^hijklmn^	49.65	24
GAC	18.09 ± 0.38 ^ghijk^	17.48 ± 1.04 ^b^	14.80 ± 0.59 ^cd^	185.27 ± 27.39 ^hijkl^	35.14 ± 0.37 ^klmno^	64.33	8
GN	16.44 ± 0.57 ^klmn^	10.72 ± 0.23 ^lmn^	4.85 ± 1.16 ^pqr^	208.48 ± 30.42 ^efghijkl^	36.08 ± 2.81 ^jklmn^	46.19	27
GOC	19.58 ± 0.50 ^efg^	13.46 ± 0.51 ^efghij^	11.12 ± 0.30 ^efghi^	163.88 ± 20.46 ^jkl^	42.45 ± 1.68 ^defg^	57.37	15
HMR	24.20 ± 0.69 ^b^	20.40 ± 0.24 ^a^	17.23 ± 0.29 ^b^	270.31 ± 13.88 ^cdefg^	51.74 ± 0.27 ^ab^	82.81	3
HY	15.20 ± 0.60 ^mno^	14.47 ± 0.60 ^defgh^	8.67 ± 0.28 ^jklm^	196.63 ± 16.56 ^ghijkl^	33.55 ± 0.14 ^nop^	53.40	21
KZJ22	21.11 ± 1.05 ^cde^	14.92 ± 0.91 ^cdefg^	12.51 ± 1.15 ^def^	357.54 ± 0.53 ^b^	43.78 ± 1.07 ^def^	71.69	4
MBWD	13.13 ± 0.18 ^pq^	9.17 ± 0.94 ^nopq^	4.92 ± 0.45 ^pqr^	178.20 ± 22.21 ^ijkl^	25.62 ± 0.50 ^rs^	38.01	32
MTH	21.65 ± 0.65 ^c^	14.77 ± 0.68 ^cdefg^	12.82 ± 0.48 ^de^	231.29 ± 39.15 ^defghij^	41.82 ± 1.70 ^efgh^	64.25	9
MXG	14.86 ± 0.40 ^no^	13.04 ± 0.25 ^ghijk^	9.98 ± 0.96 ^ghijk^	75.78 ± 35.22 ^m^	24.50 ± 0.86 s	42.64	30
NG20	19.30 ± 0.11 ^fgh^	13.39 ± 0.64 ^efghij^	9.43 ± 0.55 ^ijkl^	288.99 ± 3.61 ^bcd^	37.61 ± 0.26 ^hijklmn^	59.76	13
NH	20.60 ± 0.35 ^cdef^	16.50 ± 0.27 ^bcd^	12.82 ± 0.73 ^de^	213.25 ± 26.59 ^efghijkl^	45.70 ± 1.07 ^cde^	67.14	5
OG	19.04 ± 0.60 ^fghi^	11.44 ± 0.83 ^jklm^	9.59 ± 0.49 ^hijk^	249.79 ± 21.33 ^cdefghi^	41.04 ± 0.60 ^efghi^	57.08	16
PG	18.36 ± 0.22 ^ghij^	14.39 ± 0.37 ^defgh^	11.31 ± 1.08 ^efghi^	250.70 ± 42.77 ^cdefghi^	39.87 ± 1.39 ^fghijk^	62.16	11
PTY	17.20 ± 0.76 ^jkl^	15.54 ± 0.58 ^bcde^	7.71 ± 0.45 ^klmno^	201.56 ± 18.04 ^fghijkl^	34.19 ± 0.78 ^mno^	54.12	19
QJ	21.45 ± 0.54 ^c^	16.36 ± 0.06 ^bcd^	10.36 ± 0.73 ^fghij^	256.04 ± 37.59 ^cdefgh^	46.81 ± 2.56 ^cd^	66.88	6
QOG	18.29 ± 0.36 ^ghij^	10.62 ± 0.34 ^lmn^	8.42 ± 0.59 ^jklmn^	277.70 ± 15.99 ^cde^	40.36 ± 0.96 ^fghij^	55.89	17
RN1	16.33 ± 0.48 ^lmn^	12.41 ± 0.95 ^hijkl^	8.52 ± 0.71 ^jklm^	214.83 ± 20.09 ^defghijkl^	30.71 ± 1.99 ^opq^	50.41	23
SJY	12.18 ± 0.32 ^pq^	7.80 ± 0.55 ^pq^	3.87 ± 0.07 ^rs^	149.67 ± 4.81 ^lm^	24.34 ± 0.32 ^s^	33.03	34
SW	21.32 ± 0.60 ^cd^	13.24 ± 0.92 ^fghij^	10.39 ± 0.76 ^fghij^	242.22 ± 28.50 ^defghi^	40.02 ± 1.70 ^fghij^	59.31	14
SYXX	16.85 ± 0.52 ^jklm^	11.74 ± 0.81 ^ijklm^	8.35 ± 0.83 ^jklmn^	209.35 ± 24.08 ^efghijkl^	36.65 ± 1.81 ^ijklmn^	51.83	22
TC	24.80 ± 0.55 ^b^	19.94 ± 0.30 ^a^	16.88 ± 1.60 ^bc^	468.96 ± 34.84 ^a^	49.16 ± 2.51 ^bc^	91.26	2
TCH	16.82 ± 0.31 ^jklm^	10.04 ± 0.50 ^1mno^	5.57 ± 0.24 ^opqr^	227.70 ± 11.03 ^defghijk^	38.84 ± 0.88 ^ghijklm^	48.49	25
WHOG	17.64 ± 0.37 ^hijkl^	11.02 ± 0.23 ^klmn^	7.72 ± 0.62 ^klmno^	256.28 ± 28.81 ^cdefgh^	39.12 ± 2.01 ^fghijkl^	53.85	20
WZ	17.66 ± 0.14 ^hijkl^	11.77 ± 0.53 ^ijklm^	9.81 ± 0.53 ^hijk^	210.70 ± 16.96 ^efghijkl^	40.07 ± 1.88 ^fghij^	55.20	18
YHY	13.57 ± 0.17 ^op^	9.76 ± 0.63 ^mnop^	6.05 ± 0.53 ^nopqr^	158.18 ± 8.30 ^jkl^	26.28 ± 1.21 ^qrs^	39.28	31
YL	17.57 ± 0.99 ^ijkl^	13.75 ± 1.56 ^efghij^	9.22 ± 0.89 ^ijkl^	275.85 ± 27.24 ^cdef^	41.02 ± 0.88 ^efghi^	60.81	12
YXC	15.49 ± 0.73 ^mn^	7.89 ± 0.80 ^opq^	6.64 ± 0.04 ^mnopq^	188.26 ± 8.62 ^hijkl^	34.69 ± 0.80 ^lmno^	43.16	29
ZXY	11.52 ± 0.03 ^q^	7.40 ± 0.13 ^qr^	4.70 ± 0.08 ^qr^	158.83 ± 17.20 ^jkl^	24.44 ± 2.01 ^s^	34.05	33

Results were the mean ± SD (*n* = 3) on a dried weight (g) of citrus basis. Total phenolics were calculated as mg gallic acid equivalents (GAE)/g DW. Antioxidant capacities (DPPH, FRAP, ORAC and CUPRAC) were calculated as mg trolox equivalent antioxidant capacities (TEAC)/g DW. Values within each column followed by different superscript letters were significantly different (*p* < 0.05) according to Tukey’s tests. * Antioxidant index score = [(sample score/best score) × 100], averaged for all four tests for each cultivar for the antioxidant potency composite (APC) index. ^#^ Ranked according to the APC index.

**Table 3:** Total phenolics and antioxidant properties of citrus fruit albedo of 35 cultivars.

Cultivars	Total Phenolics	DPPH	FRAP	ORAC	CUPRAC	APC Index *	Rank ^#^
AY27	15.19 ± 0.73 ^fghijklm^	8.22 ± 0.38 ^defghi^	15.01 ± 0.08 ^d^	55.49 ± 7.65 ^m^	18.30 ± 0.83 ^mnop^	38.40	22
AY30	19.84 ± 1.30 ^bcd^	16.53 ± 0.49 ^a^	18.45 ± 0.88 ^c^	288.00 ± 39.86 ^efg^	36.77 ± 0.89 ^cd^	72.09	2
AY31	11.68 ± 1.28 ^lmn^	4.73 ± 0.35 ^kl^	8.24 ± 0.68 ^ijkl^	73.31 ± 2.11 ^lm^	16.26 ± 0.55 ^opq^	26.31	33
BZH	14.45 ± 2.04 ^ghijklmn^	10.62 ± 1.57 ^cd^	11.64 ± 0.92 ^efg^	220.49 ± 24.87 ^ghijk^	31.25 ± 1.49 ^efg^	51.52	11
CR	13.46 ± 0.15 ^ijklmn^	5.88 ± 0.74 ^ijkl^	7.28 ± 0.27 ^jklmno^	232.58 ± 11.48 ^ghij^	17.42 ± 0.21 ^nop^	33.49	26
CX	10.35 ± 0.48 ^n^	4.37 ± 0.69 ^l^	5.68 ± 0.24 ^lmno^	177.82 ± 13.67 ^hijkl^	22.21 ± 1.10 ^jklm^	30.33	32
DF	13.13 ± 0.44 ^jklmn^	5.00 ± 0.36 ^jkl^	7.12 ± 0.03 ^jklmno^	417.66 ± 22.58 ^cd^	22.77 ± 0.44 ^jkl^	41.79	20
GAC	27.15 ± 3.53 ^a^	17.43 ± 0.93 ^a^	25.26 ± 2.77 ^a^	420.79 ± 53.79 ^cd^	39.34 ± 3.01 ^bc^	86.39	1
GN	15.74 ± 1.16 ^defghijkl^	7.00 ± 0.81 ^fghijk^	7.24 ± 0.34 ^jklmno^	429.84 ± 62.99 ^cd^	26.20 ± 1.09 ^hij^	47.04	15
GOC	16.12 ± 1.71 ^defghijk^	6.97 ± 0.57 ^fghijk^	8.06 ± 0.88 ^jkl^	382.81 ± 24.59 ^de^	23.24 ± 0.15 ^jk^	44.48	16
HMR	17.78 ± 1.65 ^cdefgh^	11.65 ± 0.26 ^bc^	14.50 ± 0.44 ^d^	358.36 ± 33.63 ^def^	33.35 ± 0.31 ^def^	62.03	7
HY	17.59 ± 0.15 ^cdefg^	8.60 ± 1.49 ^defg^	6.80 ± 0.51 ^jklmno^	424.62 ± 24.53 ^cd^	25.56 ± 0.60 ^ij^	48.33	14
KZJ22	16.39 ± 0.63 ^defghij^	6.90 ± 0.67 ^fghijk^	11.84 ± 0.46 ^ef^	245.27 ± 4.35 ^g^	23.53 ± 0.75 ^opq^	43.21	17
MBWD	13.82 ± 0.27 ^hijklmn^	7.37 ± 0.67 ^fghij^	8.02 ± 0.19 ^jkl^	206.45 ± 15.84 ^ghijk^	14.13 ± 2.06 ^pqr^	33.60	25
MTH	18.96 ± 0.50 ^cdef^	13.92 ± 0.55 ^b^	17.77 ± 1.23 ^c^	215.75 ± 28.81 ^ghijk^	34.77 ± 1.47 ^de^	63.98	6
MXG	14.97 ± 2.42 ^fghijklm^	5.13 ± 0.06 ^jkl^	10.71 ± 0.64 ^fg^	177.63 ± 2.90 ^hijkl^	12.12 ± 0.39 ^qr^	30.94	31
NG20	15.35 ± 0.27 ^fghijklm^	4.86 ± 0.43 ^jkl^	9.22 ± 0.17 ^ghij^	176.15 ± 30.27 ^hijkl^	20.09 ± 0.61 ^klmno^	33.32	27
NH	13.73 ± 0.57 ^hijklmn^	6.55 ± 0.33 ^ghijkl^	7.67 ± 0.19 ^jklm^	116.37 ± 1.22 ^klm^	22.21 ± 1.39 ^jklm^	33.12	29
OG	20.98 ± 0.60 ^bc^	8.96 ± 1.10 ^defg^	13.28 ± 1.25 ^de^	549.63 ± 73.64 ^b^	46.43 ± 1.98 ^a^	71.10	4
PG	15.49 ± 0.57 ^efghijklm^	17.65 ± 0.26 ^a^	21.56 ± 1.56 ^b^	179.13 ± 2.73 ^hijkl^	34.96 ± 2.78 ^de^	71.77	3
PTY	24.00 ± 1.50 ^ab^	12.28 ± 0.33 ^bc^	11.57 ± 0.34 ^efgh^	508.19 ± 85.71 ^bc^	33.86 ± 1.85 ^def^	65.82	5
QJ	15.49 ± 0.72 ^efghijklm^	10.23 ± 0.82 ^cde^	11.51 ± 0.36 ^efgh^	198.88 ± 12.12 ^ghijk^	28.14 ± 0.85 ^g^	48.37	13
QOG	19.69 ± 1.54 ^cde^	8.49 ± 0.86 ^defgh^	10.65 ± 0.56 ^fg^	404.23 ± 4.79 ^cd^	42.67 ± 1.95 ^ab^	60.45	8
RN1	14.18 ± 1.50 ^higklmn^	8.04 ± 0.78 ^efghi^	11.89 ± 0.52 ^ef^	126.91 ± 13.19 ^jklm^	20.40 ± 0.25 ^klmno^	38.82	21
SJY	14.37 ± 1.23 ^higklmn^	7.31 ± 0.74 ^fghij^	7.53 ± 0.33 ^jklmn^	224.79 ± 19.36 ^ghij^	12.41 ± 1.49 ^qr^	32.78	30
SW	15.54 ± 0.81 ^efghijklm^	7.35 ± 0.99 ^fghij^	9.12 ±0.83 ^ghij^	412.33 ± 29.49 ^cd^	26.11 ± 0.83 ^hij^	48.70	12
SYXX	13.41 ± 1.48 ^ijklmn^	5.22 ± 0.73 ^jkl^	6.51 ± 0.29 ^klmno^	360.47 ± 49.12 ^de^	18.66 ± 1.6l ^mno^	37.18	23
TC	16.12 ± 1.82 ^defghijk^	8.56 ± 0.66 ^defg^	9.04 ± 0.48 ^hijk^	677.90 ± 51.50 ^a^	22.61 ± 0.27 ^jkl^	58.25	9
TCH	11.37 ± 1.58 ^mn^	4.75 ± 0.44 ^kl^	5.42 ± 0.60 ^mno^	253.81 ± 16.73 ^fg^	22.83 ± 1.49 ^jkl^	33.75	24
WHOG	18.67 ± 1.05 ^cdefg^	8.59 ± 0.44 ^defg^	10.66 ± 0.42 ^fg^	282.05 ± 78.31 ^efgh^	41.25 ± 1.21 ^b^	55.33	10
WZ	11.68 ± 1.24 ^lmn^	4.72 ± 0.18 ^kl^	6.33 ± 0.56 ^lmno^	248.27 ± 30.14 ^g^	20.81 ± 0.62 ^klmn^	33.31	28
YHY	16.85 ± 0.57 ^cdefghij^	4.56 ± 2.19 ^kl^	4.82 ± 0.37 ^o^	198.22 ± 10.99 ^ghijk^	11.92 ± 1.59 ^r^	24.96	35
YL	13.91 ± 0.64 ^higklmn^	5.92 ± 0.17 ^ijkl^	7.31 ± 0.84 ^jklmno^	436.49 ± 14.06 ^cd^	21.00 ± 0.18 ^klmn^	43.02	19
YXC	12.06 ± 1.41 ^klmn^	9.11 ± 0.70 ^def^	11.30 ± 1.01 ^efgh^	76.45 ± 8.52 ^lm^	30.19 ± 1.02 ^fgh^	43.16	18
ZXY	14.00 ± 0.39 ^higklmn^	6.09 ± 0.74 ^hijkl^	5.07 ± 0.70 ^no^	168.30 ± 11.89 ^ijkl^	11.34 ± 1.08 ^r^	25.96	34

Results were the mean ± SD (*n* = 3) on a dried weight (g) of citrus basis. Total phenolics were calculated as mg gallic acid equivalents (GAE)/g DW. Antioxidant capacities (DPPH, FRAP, ORAC and CUPRAC) were calculated as mg trolox equivalent antioxidant capacities (TEAC)/g DW. Values within each column followed by different superscript letters were significantly different (*p* < 0.05) according to Tukey’s tests. * Antioxidant index score = [(sample score/best score) × 100], averaged for all four tests for each cultivar for the antioxidant potency composite (APC) index. ^#^ Ranked according to the APC index.

**Table 4:** Total phenolics and antioxidant properties of citrus fruit segment membrane of 35 cultivars.

Cultivars	Total Phenolics	DPPH	FRAP	ORAC	CUPRAC	APC Index *	Rank ^#^
AY27	8.84 ± 0.10 ^cdefgh^	5.12 ± 0.53 ^defghijk^	15.92 ± 0.77 ^cd^	49.03 ± 7.40 ^pq^	5.03 ± 0.20 ^def^	63.12	18
AY30	8.59 ± 0.21 ^efgh^	6.84 ± 0.98 ^ab^	19.13 ± 0.70 ^ab^	102.13 ± 6.06 ^jklmn^	6.13 ± 0.09 ^ab^	83.70	4
AY31	7.84 ± 0.38 ^hi^	3.15 ± 0.29 ^opq^	10.60 ± 0.08 ^klm^	69.27 ± 5.44 ^nopq^	3.16 ± 0.23 ^lmn^	44.79	33
BZH	8.90 ± 0.16 ^cdefgh^	5.61 ± 0.15 ^cdef^	15.41 ± 0.28 ^cde^	129.18 ± 10.93 ^fghijk^	4.77 ± 0.26 ^defghi^	72.72	8
CR	8.18 ± 0.67 ^gh^	3.62 ± 0.38 ^lmnopq^	9.40 ± 0.91 ^mno^	98.24 ± 3.84 ^klmno^	4.05 ± 0.33 ^ghijkl^	51.83	30
CX	6.27 ± 0.29 ^j^	3.55 ± 0.03 ^mnopq^	8.30 ± 0.27 ^o^	77.50 ± 5.45 ^lmnop^	2.57 ± 0.17 ^n^	41.92	34
DF	8.71 ± 0.18 ^defgh^	5.25 ± 0.50 ^defghi^	11.69 ± 0.07 ^ijkl^	132.53 ± 8.86 ^efghijk^	3.91 ± 0.30 ^ijklm^	63.83	17
GAC	12.56 ± 0.70 ^a^	5.73 ± 0.12 ^bcde^	19.83 ± 0.90 ^a^	183.38 ± 5.16 ^ab^	6.24 ± 0.34 ^ab^	90.96	1
GN	9.70 ± 0.32 ^bcdef^	3.67 ± 0.17 ^lmnopq^	9.96 ± 0.16 ^lmno^	164.49 ± 10.28 ^bcdef^	3.30 ± 0.23 ^klmn^	57.70	25
GOC	9.44 ± 0.43 ^bcdefg^	5.27 ± 0.17 ^defgh^	12.83 ± 0.49 ^ghij^	139.82 ± 14.40 ^defghi^	3.89 ± 0.25 ^ijklm^	66.13	12
HMR	10.63 ± 0.33 ^b^	7.34 ± 0.39 ^a^	17.32 ± 0.13 ^ab^	133.80 ± 7.90 ^efghijk^	5.45 ± 0.21 ^bcd^	84.24	3
HY	9.88 ± 0.74 ^bcde^	4.80 ± 0.52 ^efghijkl^	13.53 ± 0.89 ^efghi^	171.10 ± 19.05 ^bcd^	6.37 ± 0.18 ^a^	78.89	5
KZJ22	8.55 ± 0.60 ^efgh^	2.96 ± 0.22 ^pq^	10.39 ± 0.18 ^klmn^	98.58 ± 4.77 ^jklmno^	3.01 ± 0.17 ^mn^	46.79	32
MBWD	8.67 ± 0.12 ^defgh^	4.55 ± 0.55 ^efghijklmn^	8.50 ± 0.94 ^no^	147.93 ± 12.91 ^cdefg^	4.60 ± 0.46 ^defghi^	61.97	19
MTH	7.64 ± 0.34 ^hij^	5.73 ± 0.05 ^bcde^	13.26 ± 0.65 ^ghi^	72.25 ± 5.65 ^mnopq^	4.13 ± 0.08 ^ghijk^	61.09	20
MXG	7.77 ± 0.25 ^hi^	4.34 ± 0.15 ^hijklmno^	15.78 ± 0.92 ^cd^	59.10 ± 6.67 ^pq^	4.85 ± 0.29 ^defgh^	60.78	21
NG20	8.84 ± 0.37 ^cdefgh^	3.42 ± 0.47 ^nopq^	9.96 ± 0.24 ^lmno^	139.79 ± 19.17 ^defghi^	3.43 ± 0.03 ^jklmn^	54.40	28
NH	9.55 ± 0.63 ^bcdefg^	4.50 ± 0.54 ^fghijklmn^	14.00 ± 0.35 ^defgh^	111.73 ± 2.57 ^hijkl^	4.64 ± 0.23 ^defghi^	64.56	16
OG	10.63 ± 0.56 ^b^	5.29 ± 0.34 ^defgh^	10.80 ± 0.42 ^jklm^	141.78 ±12.77 ^defgh^	2.73 ± 0.09 ^n^	59.32	23
PG	7.72 ± 0.38 ^hij^	6.31 ± 0.05 ^abcd^	14.59 ± 0.83 ^defg^	71.47 ± 6.54 ^mnopq^	4.23 ± 0.04 ^fghij^	65.04	14
PTY	9.66 ± 0.46 ^bcdef^	3.91 ± 0.17 ^klmopq^	14.86 ± 0.79 ^defg^	133.13 ± 6.13 ^efghijk^	4.60 ± 0.17 ^defghi^	66.04	13
QJ	8.70 ± 0.39 ^defgh^	5.64 ± 0.30 ^bcdef^	13.33 ± 0.13 ^fghi^	104.83 ± 9.21 ^ijklm^	5.12 ± 1.03 ^def^	68.66	10
QOG	9.06 ± 0.71 ^cdefgh^	4.44 ± 0.47 ^fghijklmn^	9.52 ± 0.35 ^mno^	123.99 ± 3.26 ^ghijk^	3.07 ± 0.13 ^mn^	54.01	29
RN1	8.90 ± 0.58 ^cdefgh^	3.95 ± 0.25 ^jklmnop^	11.64 ± 0.71 ^ijkl^	106.21 ± 13.69 ^ijklm^	3.98 ± 0.28 ^hijkl^	56.46	27
SJY	8.49 ± 0.57 ^efgh^	3.52 ± 0.43 ^mnopq^	9.78 ± 0.63 ^lmno^	178.73 ± 28.31 ^abc^	4.88 ± 0.12 ^defg^	64.86	15
SW	9.01 ± 0.13 ^cdefgh^	5.57 ± 0.51 ^cdefg^	14.63 ± 1.00 ^defg^	152.56 ± 10.44 ^bcdefg^	4.42 ± 0.09 ^efghi^	73.02	7
SYXX	9.70 ± 0.18 ^bcdef^	3.50 ± 0.33 ^mnopq^	9.51 ± 0.27 ^mno^	166.46 ± 2.43 ^bcde^	4.00 ± 0.07 ^ghijkl^	59.53	22
TC	9.58 ± 0.69 ^bcdefg^	6.64 ± 0.53 ^abc^	18.86 ± 1.38 ^ab^	170.14 ± 2.32 ^bcd^	6.02 ± 0.08 ^abc^	90.38	2
TCH	6.42 ± 0.17 ^ij^	2.72 ± 0.18 ^q^	9.37 ± 0.27 ^mno^	64.69 ± 9.16 ^opq^	2.87 ± 0.10 ^n^	40.08	35
WHOG	8.44 ± 0.47 ^efgh^	4.12 ± 0.33 ^hijklmnop^	9.50 ± 0.83 ^mno^	101.30 ± 10.04 ^jklmn^	3.06 ± 0.10 ^mn^	50.14	31
WZ	8.36 ± 0.57 ^fgh^	4.65 ± 0.24 ^efghijklm^	10.00 ± 0.71 ^lmno^	131.91 ± 17.31 ^efghijk^	3.36 ± 0.03 ^jklmn^	57.42	26
YHY	10.10 ± 0.34 ^bcd^	4.06 ± 0.31 ^ijklmnop^	10.82 ± 0.61 ^jklm^	208.86 ± 0.33 ^a^	5.13 ± 0.12 ^cde^	72.60	9
YL	10.20 ± 0.24 ^bc^	4.38 ± 0.21 ^ghijklmn^	12.24 ± 0.28 ^hijk^	153.20 ± 25.10 ^bcdefg^	4.63 ± 0.28 ^defghi^	66.86	11
YXC	6.66 ± 0.43 ^ij^	4.82 ± 0.47 ^efghijkl^	15.33 ± 0.52 ^cdef^	37.26 ± 3.92 ^q^	4.66 ± 0.43 ^defghi^	58.49	24
ZXY	8.96 ± 0.61 ^cdefgh^	5.14 ± 0.23 ^defghij^	9.95 ± 0.10 ^lmno^	179.93 ± 8.53 ^abc^	6.30 ± 0.19 ^ab^	76.31	6

Results were the mean ± SD (*n* = 3) on a dried weight (g) of citrus basis. Total phenolics were calculated as mg gallic acid equivalents (GAE)/g DW. Antioxidant capacities (DPPH, FRAP, ORAC and CUPRAC) were calculated as mg trolox equivalent antioxidant capacities (TEAC)/g DW. Values within each column followed by different superscript letters were significantly different (*p* < 0.05) according to Tukey’s tests. * Antioxidant index score = [(sample score/best score) × 100], averaged for all four tests for each cultivar for the antioxidant potency composite (APC) index. ^#^ Ranked according to the APC index.

**Table 5:** Total phenolics and antioxidant properties of citrus fruit juice sacs of 35 cultivars.

Cultivars	Total Phenolics	DPPH	FRAP	ORAC	CUPRAC	APC Index *	Rank ^#^
AY27	6.35 ± 0.13 ^a^	4.73 ± 0.15 ^bcd^	13.00 ± 1.09 ^a^	62.32 ± 7.68 ^bc^	5.01 ± 0.27 ^cde^	84.23	1
AY30	6.10 ± 0.20 ^abc^	5.02 ± 0.36 ^abc^	8.54 ± 0.37 ^defgh^	32.26 ± 2.36 ^ghijk^	6.04 ± 0.20 ^ab^	70.68	8
AY31	4.75 ± 0.21 ^ghijk^	2.09 ± 0.27 ^opq^	7.42 ± 0.56 ^ghijkl^	40.59 ± 2.64 ^efg^	2.85 ± 0.16 ^mno^	47.04	31
BZH	5.25 ± 0.13 ^defghij^	4.29 ± 0.18 ^cdefgh^	8.36 ± 0.43 ^defghij^	20.35 ± 0.70 ^l^	6.32 ± 1.08 ^ab^	64.22	12
CR	5.84 ± 0.21 ^abcdef^	4.54 ± 0.12 ^cdefg^	8.27 ± 0.39 ^defghij^	53.06 ± 4.85 ^cd^	4.90 ± 0.20 ^de^	70.85	7
CX	4.18 ± 0.10 ^k^	1.66 ± 0.03 ^pq^	4.28 ± 0.34 ^o^	27.56 ± 1.35 ^ijkl^	2.51 ± 0.04 ^no^	33.60	34
DF	5.89 ± 0.04 ^abcde^	3.74 ± 0.28 ^ghijk^	7.07 ± 0.55 ^hijklm^	38.83 ± 2.42 ^efgh^	3.97 ± 0.10 ^fghijkl^	57.01	20
GAC	6.06 ± 0.39 ^abcd^	4.41 ± 0.18 ^cdefg^	11.96 ± 0.53 ^ab^	54.12 ± 4.41 ^cd^	4.58 ± 0.20 ^defghi^	76.58	5
GN	5.27 ± 0.05 ^cdefghij^	3.58 ± 0.26 ^hijk^	6.60 ± 0.28 ^jklmn^	28.76 ± 1.54 ^hijkl^	4.53 ± 0.51 ^defghij^	54.09	26
GOC	5.42 ± 0.18 ^bcdefghi^	3.80 ± 0.21 ^fghij^	6.74 ± 0.46 ^ijklmn^	29.49 ± 1.13 ^ghijkl^	3.87 ± 0.23 ^ghijkl^	53.19	28
HMR	5.27 ± 0.15 ^cdefghij^	3.23 ± 0.08 ^ijkl^	7.45 ± 0.61 ^ghijkl^	28.78 ± 1.70 ^hijkl^	4.47 ± 0.05 ^defghij^	53.99	27
HY	5.98 ± 0.31 ^abcd^	5.40 ± 0.21 ^ab^	10.04 ± 0.65 ^cd^	27.64 ± 0.39 ^hijkl^	6.96 ± 0.09 ^a^	77.00	4
KZJ22	5.70 ± 0.29 ^abcdef^	1.46 ± 0.15 ^q^	5.63 ± 0.33 ^mno^	75.63 ± 3.81 ^a^	2.30 ± 0.19 ^o^	50.46	30
MBWD	4.80 ± 0.14 ^ghijk^	4.22 ± 0.20 ^cdefgh^	7.61 ± 0.29 ^ghijkl^	20.15 ± 3.74 ^l^	4.53 ± 0.19 ^defghij^	55.98	22
MTH	5.39 ± 0.39 ^bcdefghi^	3.58 ± 0.15 ^hijk^	8.36 ± 0.43 ^defghij^	35.82 ± 3.47 ^ghi^	3.88 ± 0.27 ^ghijkl^	57.47	19
MXG	5.02 ± 0.11 ^fghij^	2.41 ± 0.11 ^mnop^	7.43 ± 0.34 ^ghijkl^	48.81 ± 3.20 ^de^	3.17 ± 0.30 ^lmno^	52.32	29
NG20	5.44 ± 0.03 ^bcdefghi^	4.25 ± 0.18 ^cdefgh^	11.9 ± 1.19 ^ab^	26.84 ± 0.92 ^ijkl^	4.77 ± 0.14 ^defg^	67.43	9
NH	6.39 ± 0.42 ^a^	4.74 ± 0.51 ^bcd^	9.13 ± 1.08 ^cdefg^	57.02 ± 2.10 ^bcd^	5.87 ± 0.13 ^bc^	78.17	3
OG	6.18 ± 0.39 ^ab^	3.25 ± 0.15 ^ijkl^	5.97 ± 0.81 ^lmno^	55.16 ± 7.92 ^bcd^	3.62 ± 0.20 ^jklm^	56.90	21
PG	6.00 ± 0.23 ^abcd^	4.17 ± 0.16 ^defgh^	10.32 ± 0.46 ^bc^	36.58 ± 1.61 ^ghi^	4.36 ± 0.23 ^defghij^	65.79	10
PTY	5.84 ± 0.11 ^abcedf^	4.28 ± 0.27 ^cdefgh^	7.57 ± 0.29 ^ghijkl^	60.32 ± 7.35 ^bc^	5.02 ± 0.29 ^cde^	71.20	6
QJ	5.10 ± 0.26 ^efghij^	4.68 ± 0.22 ^bcde^	7.82 ± 0.14 ^fghijk^	22.59 ± 1.15 ^kl^	5.10 ± 0.30 ^cd^	61.24	14
QOG	5.91 ± 0.35 ^abcde^	2.68 ± 0.36 ^lmno^	6.74 ± 0.12 ^ijklmn^	48.80 ± 2.53 ^de^	4.14 ± 0.15 ^efghijk^	55.66	23
RN1	4.73 ± 0.42 ^ghijk^	3.31 ± 0.15 ^ijkl^	9.04 ± 0.21 ^cdefg^	37.03 ± 4.50 ^fghi^	4.15 ± 0.01 ^efghijk^	58.97	16
SJY	4.62 ± 0.15 ^ijk^	4.57 ± 0.09 ^cdef^	8.44 ± 0.39 ^defghi^	26.43 ± 4.43 ^ijkl^	4.81 ± 0.31 ^def^	62.18	13
SW	5.90 ± 0.42 ^abcde^	4.01 ± 0.20 ^defghi^	7.95 ± 0.05 ^efghijk^	35.24 ± 0.94 ^ghij^	4.63 ± 0.10 ^defghi^	61.06	15
SYXX	5.49 ± 0.10 ^bcdefgh^	3.51 ± 0.26 ^hijk^	6.18 ± 0.24 ^klmn^	53.77 ± 1.61 ^cd^	3.80 ± 0.18 ^ijkl^	58.62	17
TC	6.20 ± 0.17 ^ab^	5.73 ± 0.62 ^a^	9.73 ± 0.58 ^cde^	35.38 ± 5.10 ^ghi^	6.54 ± 0.22 ^ab^	78.90	2
TCH	4.63 ± 0.31 ^hijk^	2.23 ± 0.26 ^nopq^	5.19 ± 0.51 ^no^	24.15 ± 1.38 ^jkl^	3.12 ± 0.20 ^lmno^	38.90	33
WHOG	6.14 ± 0.46 ^ab^	3.04 ± 0.03 ^jklm^	6.29 ± 0.02 ^klmn^	47.89 ± 1.22 ^def^	3.84 ± 0.12 ^hijkl^	54.98	24
WZ	4.67 ± 0.20 ^ghijkl^	2.98 ± 0.21 ^klmn^	5.83 ± 0.15 ^lmno^	23.84 ± 1.42 ^kl^	3.38 ± 0.04 ^klmn^	44.23	32
YHY	4.83 ± 0.13 ^ghijk^	4.61 ± 0.55 ^bcde^	9.48 ± 1.15 ^cdef^	27.52 ± 3.15 ^ijkl^	4.74 ± 0.14 ^defgh^	64.47	11
YL	5.46 ± 0.28 ^bcdefghi^	2.97 ± 0.10 ^klmn^	6.23 ± 0.57 ^klmn^	65.67 ± 2.39 ^ab^	3.29 ± 0.26 ^klmn^	58.46	18
YXC	4.36 ± 0.11 ^k^	2.79 ± 0.10 ^lmno^	7.05 ± 0.43 ^hijklm^	24.17 ± 1.26 ^jkl^	4.54 ± 0.47 ^defghij^	50.03	35
ZXY	4.53 ± 0.15 ^jk^	3.90 ± 0.23 ^efghi^	6.78 ± 0.28 ^hijklmn^	27.78 ± 1.10 ^hijkl^	4.35 ± 0.42 ^defghij^	54.86	25

Results were the mean ± SD (*n* = 3) on a dried weight (g) of citrus basis. Total phenolics were calculated as mg gallic acid equivalents (GAE)/g DW. Antioxidant capacities (DPPH, FRAP, ORAC and CUPRAC) were calculated as mg trolox equivalent antioxidant capacities (TEAC)/g DW. Values within each column followed by different superscript letters were significantly different (*p* < 0.05) according to Tukey’s tests. * Antioxidant index score = [(sample score/best score) × 100], averaged for all four tests for each cultivar for the antioxidant potency composite (APC) index. ^#^ Ranked according to the APC index.

**Table 6:** The IC_50_ value of citrus flavedo extracts treatment to three gastric cancer cell lines.

Cultivars	SGC-7901 (μg/mL)	BGC-823 (μg/mL)	AGS (μg/mL)
AY27	28.74 ± 0.61 ^abc^	22.61 ± 1.04 ^abc^	20.49 ± 0.97 ^abc^
AY30	27.50 ± 1.34 ^abc^	28.51 ± 0.81 ^abcd^	24.32 ± 1.01 ^abcd^
AY31	62.45 ± 2.49 ^efg^	68.62 ± 3.07 ^gh^	53.41 ± 0.78 ^ef^
BZH	40.65 ± 1.86 ^bcd^	38.90 ± 0.93 ^cde^	34.64 ± 1.11 ^bcd^
CR	28.50 ± 1.80 ^abc^	18.71 ± 0.82 ^a^	24.25 ± 1.32 ^abcd^
CX	80.58 ± 1.12 ^gh^	83.40 ± 5.94 ^hijk^	74.89 ± 2.43 ^gh^
DF	64.34 ± 2.31 ^efg^	42.39 ± 0.90 ^de^	36.52 ± 0.65 ^cde^
GAC	60.86 ± 1.78 ^ef^	70.37 ± 3.65 ^gh^	61.94 ± 2.18 ^fg^
GN	33.81 ± 2.01 ^abc^	35.80 ± 1.10 ^abcde^	34.51 ± 0.99 ^bcd^
GOC	55.61 ± 3.35 ^def^	49.43 ± 2.05 ^ef^	32.39 ± 1.39 ^abcd^
HMR	100.97 ± 4.35 ^i^	99.05 ± 1.97 ^k^	59.95 ± 1.34 ^fg^
HY	164.86 ± 6.31 ^k^	150.54 ± 7.45 ^l^	139.8 ± 8.13 ^j^
KZJ	39.07 ± 1.72 ^abcd^	37.80 ± 1.94 ^cde^	30.78 ± 1.55 ^abcd^
MBWD	203.51 ± 8.28 ^l^	153.78 ± 4.51 ^lm^	114.64 ± 4.46 ^i^
MTH	37.79 ± 1.71 ^abcd^	37.27 ± 1.36 ^cde^	29.34 ± 1.40 ^abcd^
MXG	89.16 ± 3.69 ^hi^	77.75 ± 4.93 ^ghij^	83.65 ± 2.86 ^h^
NG20	120.43 ± 6.27 ^j^	89.07 ± 3.92 ^ijk^	74.59 ± 2.65 ^gh^
NH	21.65 ± 0.63 ^ab^	19.88 ± 0.52 ^ab^	15.77 ± 0.66 ^a^
OG	34.60 ± 0.42 ^abc^	37.01 ± 1.37 ^bcde^	32.33 ± 0.64 ^abcd^
PG	37.76 ± 1.14 ^abcd^	33.21 ± 1.54 ^abcde^	34.02 ± 1.89 ^bcd^
PTY	207.37 ± 11.72 ^l^	192.10 ± 3.72 ^n^	165.46 ± 4.93 ^k^
QJ	20.36 ± 0.81 ^a^	24.44 ± 1.01 ^abc^	17.49 ± 0.65 ^ab^
QOG	34.39 ± 3.05 ^abc^	30.71 ± 0.63 ^abcd^	23.37 ± 0.57 ^abcd^
RN1	205.73 ± 5.71 ^l^	168.73 ± 4.87 ^m^	134.84 ± 4.77 ^j^
SJY	368.40 ± 20.35 ^o^	355.32 ± 17.47 ^p^	311.41 ± 21.54 ^m^
SW	92.66 ± 2.19 ^hi^	97.75 ± 8.99 ^k^	79.95 ± 4.45 ^h^
SYXX	81.40 ± 1.75 ^gh^	74.28 ± 3.77 ^ghi^	72.35 ± 2.11 ^gh^
TC	35.88 ± 1.39 ^abc^	33.75 ± 2.10 ^abcde^	24.24 ± 1.65 ^abcd^
TCH	69.14 ± 3.79 ^fg^	61.86 ± 2.55 ^fg^	53.82 ± 0.63 ^ef^
WHOG	26.42 ± 1.72 ^ab^	30.14 ± 1.59 ^abcd^	26.24 ± 0.93 ^abcd^
WZ	137.53 ± 6.09 ^j^	95.04 ± 1.86 ^jk^	76.47 ± 1.98 ^gh^
YHY	313.09 ± 12.31 ^n^	253.47 ± 14.60 ^o^	255.56 ± 18.56 ^l^
YL	81.39 ± 1.85 ^gh^	73.77 ± 5.29 ^ghi^	66.24 ± 0.78 ^fgh^
YXC	46.44 ± 2.30 ^cde^	34.07 ± 0.76 ^abcde^	39.32 ± 2.39 ^de^
ZXY	291.47 ± 13.15 ^m^	244.66 ± 9.86 ^o^	173.76 ± 5.97 ^k^

Results were the mean ± SD (*n* = 3) on Half inhibition rate (IC_50_). Values within each column followed by different superscript letters were significantly different (*p* < 0.05) according to Tukey’s tests.

**Table 7:** Determination of flavonoids from citrus fruits by LC-ESI-MS/MS (+ESI mode) and UPLC-DAD.

Peak No.	RT (min)	λ_max_ (nm)	[M + H]^+^ (*m*/*z*)	Formula	Fragment Ions (*m/z*)	Tentative Compounds
***Flavone C-glycosides***						
1	2.30	240.4, 330.6	595.1665	C_27_H_30_O_15_	577, 559, 541, 529, 523, 505	Vicenin-2
3	3.53	255.0, 348.0	433.1142	C_21_H_20_O_10_	415, 397, 379, 367, 337, 313	Apigenin-8-*C*-glucoside
5	4.46	272.3	463.1234	C_22_H_22_O_11_	445, 427, 397, 380, 367, 343, 313	Diosmetin-6-*C*-glucoside
***Flavanone O-glycosides***						
2	3.18	284.2	597.1823	C_27_H_32_O_15_	435, 399, 355, 289, 263, 195	Eriocitrin
4	3.61	284.2	597.1806	C_27_H_32_O_15_	433, 399, 355, 289, 263, 195	Neoeriocitrin
6	5.02	283.0, 329.4	581.186	C_27_H_32_O_14_	419, 383, 339, 273, 195, 153	Narirutin
8	5.94	283.0, 329.4	581.1865	C_27_H_32_O_14_	419, 383, 339, 315, 273, 195	Naringin
9	6.68	284.2, 331.8	611.1987	C_28_H_34_O_15_	449, 413, 369, 303, 263, 195, 153	Hesperidin
10	7.92	284.2	611.1974	C_28_H_34_O_15_	413, 369, 303, 263, 195	Neohesperidin
11	10.38	283.0	595.2019	C_28_H_34_O_14_	377, 353, 329, 287, 263, 195	Didymin
12	10.62	328.2	595.2017	C_28_H_34_O_14_	463, 379, 287, 263, 153	Poncirin
15	11.68	337.7	725.2283	C_33_H_30_O_31_	419, 404, 390, 361	Melitidin
***Flavone O-glycoside***						
7	5.65	267.0, 334.0	579.1704	C_27_H_30_O_14_	433, 315, 271, 195, 153, 127	Rhoifolin
***Polymethoxyflavonoids***						
13	11.02	215.6, 324.7	329.1019	C_18_H_16_O_6_	314, 299, 271, 228	Monohydroxytrimethoxyflavone (1)
14	11.29	328.2	359.1114	C_19_H_18_O_7_	344, 329, 314, 286, 257	Gardenin B
16	11.97	322.3	329.1016	C_18_H_16_O_6_	314, 299, 268, 136	Monohydroxytrimethoxyflavone (2)
17	12.40	343.4	331.0817	C_17_H_14_O_7_	316, 301, 273, 245, 217, 168	Trihydroxydimethoxyflavone
18	12.57	348.0	389.1227	C_20_H_20_O_8_	374, 359, 341, 298	Monohydroxy-pentamethoxyflavone (1)
19	12.84	323.5	373.1277	C_20_H_20_O_7_	358, 343, 315, 287, 181, 153	Isosinensetin
20	13.26	281.8	359.1129	C_19_H_18_O_7_	344, 329, 314, 301, 163, 147	Monohydroxytetramethoxyflavone
21	13.53	335.4	389.1227	C_20_H_20_O_8_	374, 359, 341, 298	Monohydroxypentamethoxyflavone (2)
22	13.55	351.6	403.1389	C_21_H_22_O_8_	388, 373, 359, 327, 183, 163	Hexamethoxyflavone (1)
23	14.25	240.4, 330.6	373.1289	C_20_H_20_O_7_	357, 343, 329, 312, 297, 153	Sinensetin
24	14.60	268.0, 334.0	343.118	C_19_H_18_O_6_	328, 313, 285, 257, 181, 153	Tetramethyl-*O*-isoscutellarein
25	14.80	343.4	345.0974	C_18_H_16_O_7_	330, 318	Dihydroxytrimethoxyflavone
26	14.96	210.0, 336.6	403.1392	C_21_H_22_O_8_	388, 373, 359, 327, 183, 163	Hexa-*O*-methylgossypetin
27	15.17	337.7	373.1282	C_20_H_20_O_7_	253, 211, 196, 181, 168, 150	5,7,3′,4′,5′-Pentamethoxyflavone
28	15.78	249.8, 334.2	403.1388	C_21_H_22_O_8_	388, 373, 358, 355, 327, 211, 183	Nobiletin
29	15.97	321.1	343.1175	C_19_H_18_O_6_	328, 313, 285, 257, 181, 153	Tetramethyl-*O*-scutellarein
30	16.32	350.4	375.1074	C_19_H_18_O_8_	345, 327. 197	5,4′-Dihydroxyl-3,7,8,3′-tetramethoxyflavonol
31	16.49	254.6, 341.2	433.1486	C_22_H_24_O_9_	417, 403, 388, 373, 303, 217	3,5,6,7,8,3′,4′-Heptamethoxyflavone
32	17.42	271.1, 323.5	373.1281	C_20_H_20_O_7_	358, 343, 328, 297, 211, 183	Tangeretin
33	17.82	282.0, 341.0	359.1128	C_19_H_18_O_7_	344, 329, 311, 283	6-*O*-Desmethyltangeritin/7-*O-*desmethyltangeritin
34	18.49	349.2	403.1388	C_21_H_22_O_8_	388, 373, 359, 327, 183, 163	Hexamethoxyflavone (2)
35	18.56	283.0, 341.3	389.1229	C_20_H_20_O_8_	374, 359, 341, 331, 197	5-Hydroxy-6,7,8,3′,4′-pentamethoxyflavone
36	18.99	331.8	329.1018	C_18_H_16_O_6_	299, 285, 268, 153	Monohydroxytrimethoxyflavone
37	19.36	273.5, 357.6	389.1232	C_20_H_20_O_8_	374, 359, 341, 298	Monohydroxypentamethoxyflavone (3)
38	19.65	346.9	419.1326	C_21_H_22_O_9_	404, 389, 372, 218	Natsudaidai
39	20.88	290.0, 329.4	359.1116	C_19_H_18_O_7_	344, 326, 298, 282, 255, 162	5-Hydroxy-7,8,3′,4′-tetramethoxyflavone

**Table 8:** Pearson’s correlation coefficients among total phenolics, antioxidant values and in vitro anticancer abilities in citrus flavedo.

Bioactive Capacities	Total Phenolic	DPPH	FRAP	ORAC	CUPRAC	APC Index	1/IC_50__SGC-7901_	1/IC_50 BGC-823_	1/IC_50 AGS_
Total Phenolic	1								
DPPH	0.853 **	1							
FRAP	0.888 **	0.923 **	1						
ORAC	0.735 **	0.560 **	0.563 **	1					
CUPRAC	0.936 **	0.766 **	0.787 **	0.741 **	1				
APC Index	0.958 **	0.919 **	0.930 **	0.781 **	0.916 **	1			
1/IC_50 SGC-7901_	0.513 **	0.375 *	0.421 **	0.366 **	0.590 **	0.482 **	1		
1/IC_50 BGC-823_	0.456 **	0.316	0.395 **	0.309	0.536 **	0.433 **	0.947 **	1	
1/IC_50 AGS_	0.548 **	0.392 **	0.456 **	0.396 **	0.617 **	0.514 **	0.965 **	0.949 **	1

1/IC_50_ means the reciprocal value of IC_50_; One and two asterisks represent statistical significance at *p* < 0.05 and *p* < 0.01, respectively.

**Table 9:** Pearson’s correlation coefficients among total phenolics, antioxidant values in albedo, segment membrane and juice sacs.

Bioactive Capacities	Total Phenolic	DPPH	FRAP	ORAC	CUPRAC	APC Index
**Albedo**						
Total Phenolic	1					
DPPH	0.679 **	1				
FRAP	0.645 **	0.901 **	1			
ORAC	0.500 **	0.156	0.015	1		
CUPRAC	0.656 **	0.677 **	0.626 **	0.404 **	1	
APC Index	0.804 **	0.875 **	0.806 **	0.534 **	0.876 **	1
**Segment membrane**						
Total Phenolic	1					
DPPH	0.322	1				
FRAP	0.346 *	0.741 **	1			
ORAC	0.725 **	0.086	−0.059	1		
CUPRAC	0.422 *	0.589 **	0.681 **	0.358	1	
APC Index	0.649 **	0.779 **	0.754 **	0.538 **	0.884 **	1
**Juice sacs**						
Total Phenolic	1					
DPPH	0.452 **	1				
FRAP	0.421 *	0.696 **	1			
ORAC	0.576 **	−0.183	0.033	1		
CUPRAC	0.409 *	0.896 **	0.606 **	−0.255	1	
APC Index	0.705 **	0.853 **	0.827 **	0.282	0.786 **	1

One and two asterisks represent statistical significance at *p* < 0.05 and *p* < 0.01, respectively.

## Supplementary Materials

Supplementary materials are available online.
