# Supplementary material for: Antioxidant Capacity, Anticancer Ability and Flavonoids Composition of 35 Citrus (Citrus reticulata Blanco) Varieties
Source: Molecules. 2017 Jul 5;22(7):1114. doi: 10.3390/molecules22071114 (PMC6152254; doi:10.3390/molecules22071114)
Supplement: Supplementary file 1 [file molecules-22-01114-s001.pdf]

Supplementary Materials: Figure S1–S5, Table S1–S10.

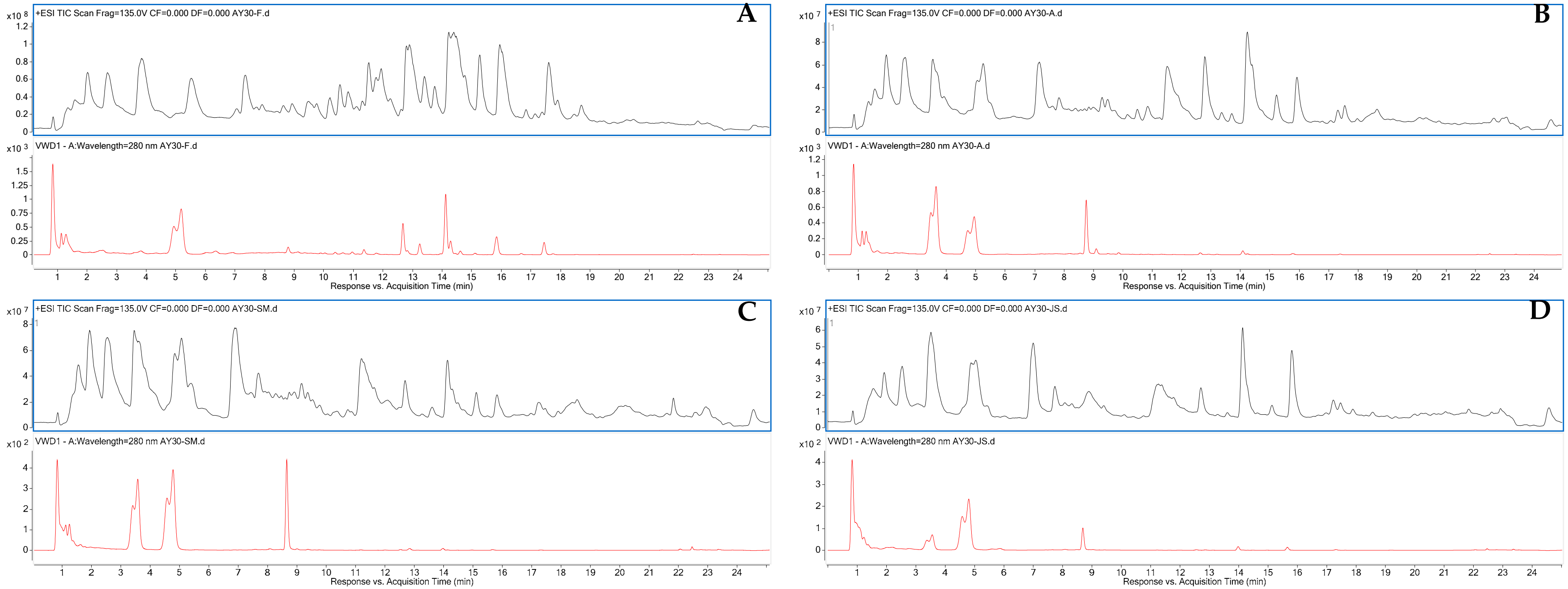

Figure S1. Typical UPLC chromatogram and mass spectrum of flavedo (**A**), albedo (**B**), segment membrane (**C**) and juice sacs (**D**) part of AY30.

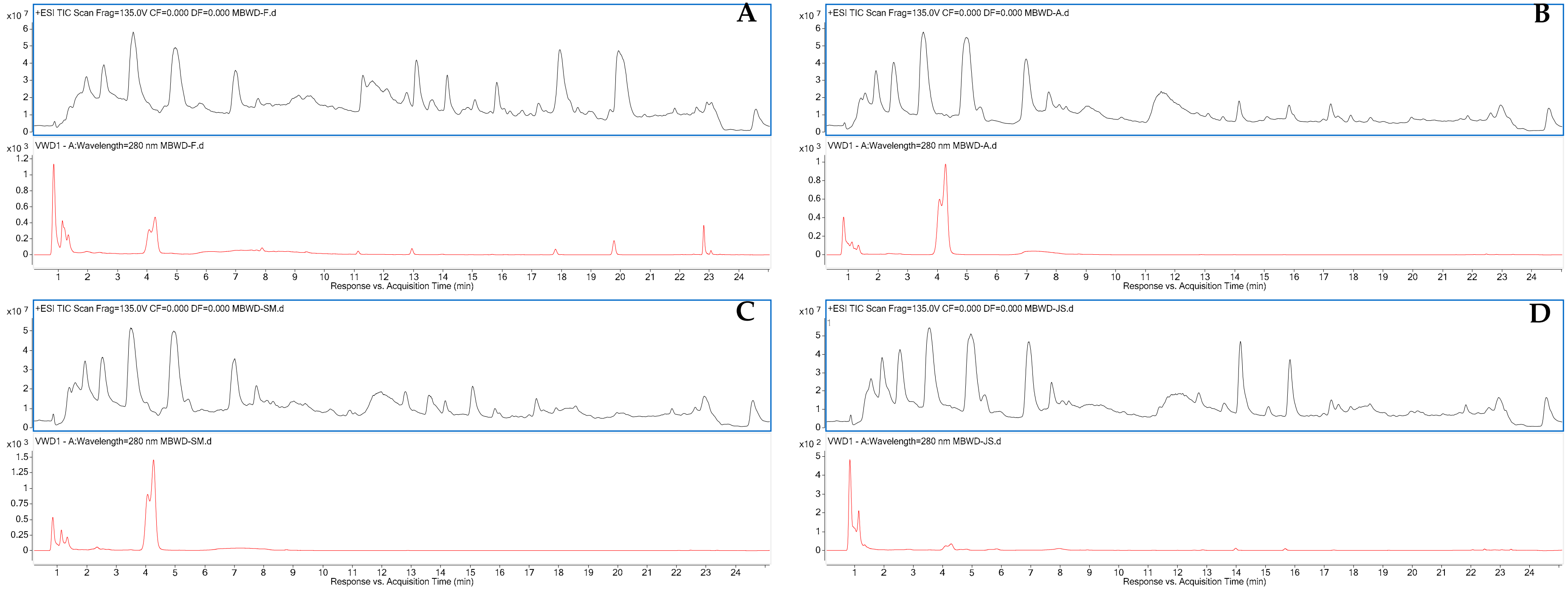

Figure S2. Typical UPLC chromatogram and mass spectrum of flavedo (**A**), albedo (**B**), segment membrane (**C**) and juice sacs (**D**) part of MBWD.

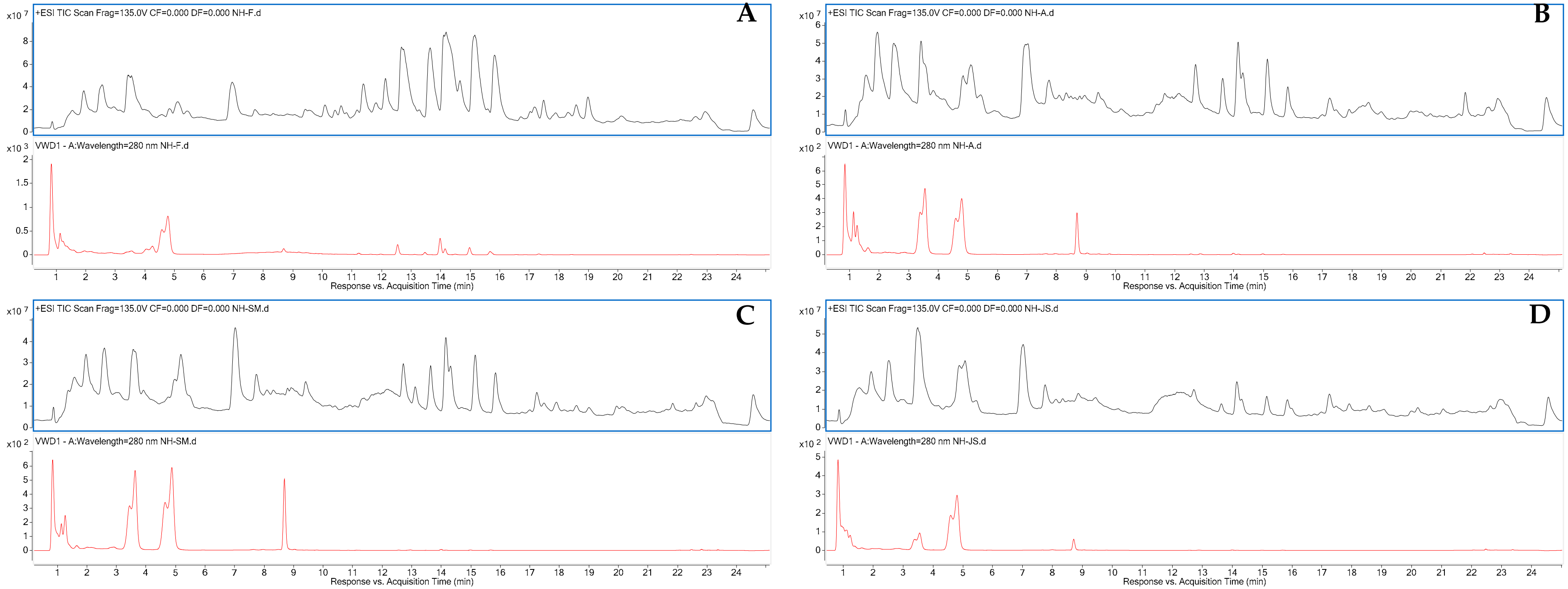

Figure S3. Typical UPLC chromatogram and mass spectrum of flavedo (**A**), albedo (**B**), segment membrane (**C**) and juice sacs (**D**) part of NH.

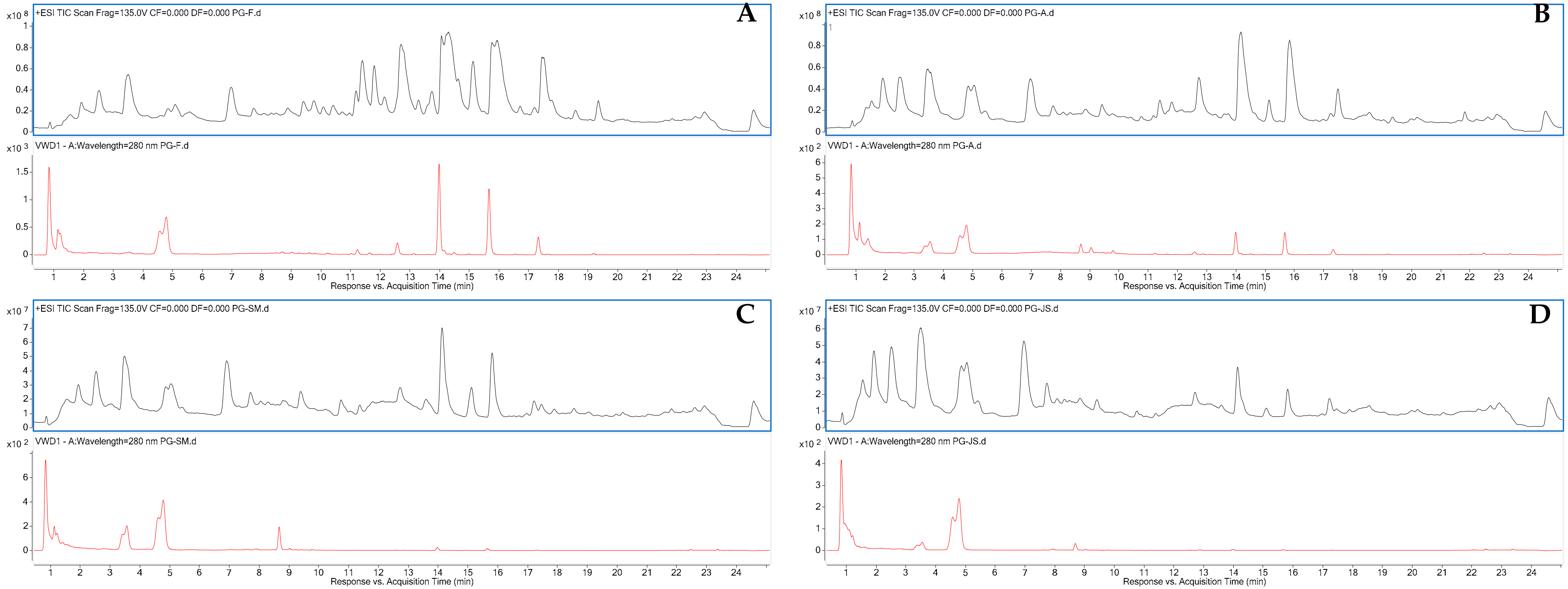

Figure S4. Typical UPLC chromatogram and mass spectrum of flavedo (**A**), albedo (**B**), segment membrane (**C**) and juice sacs (**D**) part of PG.

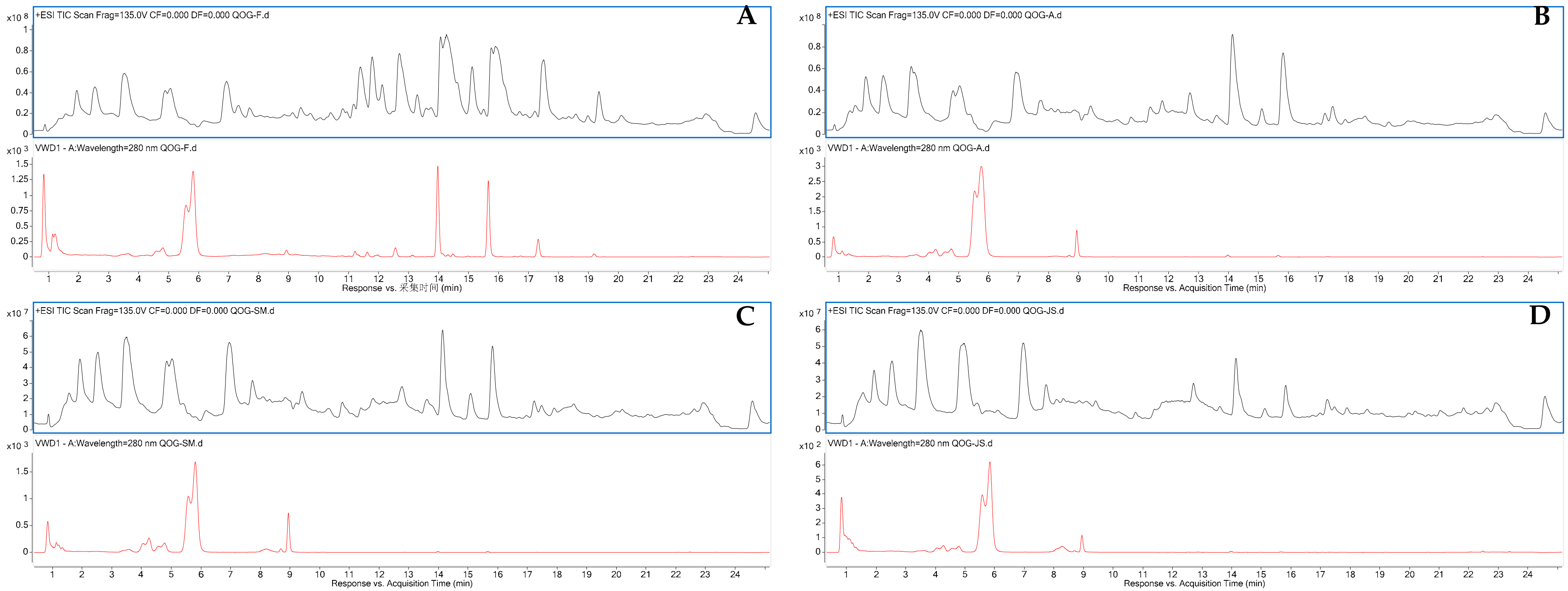

Figure S5. Typical UPLC chromatogram and mass spectrum of flavedo (**A**), albedo (**B**), segment membrane (**C**) and juice sacs (**D**) part of QOG.

Table S1. The flavone and flavanone contents (mg/g DW) in the flavedo of the 35 citrus cultivars.

Cultivars	Vicenin-2	Eriocitrin	Apigenin-8-*C*-glucoside	Neoericitrin	Diosmetin-6-*C*-glucoside	Narirutin	Rhoifolin
AY27	n.d.	n.d.	n.d.	n.d.	n.d.	1.28 ± 0.06 ^{abcde}	n.d.
AY30	n.d.	n.d.	2.76 ± 0.10 ^{c}	n.d.	n.d.	3.89 ± 0.16 ^{fgh}	n.d.
AY31	n.d.	n.d.	n.d.	n.d.	n.d.	0.84 ± 0.04 ^{abcd}	n.d.
BZH	n.d.	4.52 ± 0.13 ^{j}	n.d.	1.48 ± 0.03 ^{h}	7.24 ± 0.11 ^{c}	24.04 ± 1.24 ^{n}	n.d.
CR	n.d.	1.00 ± 0.06 ^{def}	n.d.	n.d.	n.d.	4.11 ± 0.31 ^{gh}	n.d.
CX	n.d.	0.14 ± 0.01 ^{a}	n.d.	n.d.	n.d.	4.52 ± 0.34 ^{ghi}	n.d.
DF	n.d.	0.56 ± 0.04 ^{abcd}	n.d.	0.77 ± 0.07 ^{f}	n.d.	7.21 ± 0.29 ^{jk}	n.d.
GAC	n.d.	2.20 ± 0.03 ^{h}	n.d.	1.47 ± 0.03 ^{cd}	n.d.	2.47 ± 0.23 ^{bcdefg}	n.d.
GN	n.d.	4.78 ± 0.18 ^{j}	5.25 ± 0.29 ^{d}	3.87 ± 0.09 ^{f}	n.d.	7.74 ± 0.26 ^{jk}	n.d.
GOC	n.d.	1.56 ± 0.05 ^{g}	n.d.	0.57 ± 0.04 ^{i}	n.d.	7.70 ± 0.24 ^{jk}	n.d.
HMR	n.d.	2.11 ± 0.08 ^{h}	n.d.	n.d.	n.d.	5.67 ± 0.35 ^{hij}	n.d.
HY	n.d.	0.21 ± 0.01 ^{ab}	n.d.	2.29 ± 0.19 ^{bc}	n.d.	1.11 ± 0.07 ^{abcde}	0.37 ± 0.01 ^{ab}
KZJ22	2.57 ± 0.19	1.38 ± 0.11 ^{fg}	0.99 ± 0.03 ^{a}	n.d.	n.d.	8.30 ± 0.49 ^{kl}	n.d.
MBWD	n.d.	n.d.	n.d.	n.d.	n.d.	n.d.	1.71 ± 0.09 ^{d}
MTH	n.d.	n.d.	1.57 ± 0.10 ^{b}	n.d.	n.d.	2.68 ± 0.08 ^{cdefg}	n.d.
MXG	n.d.	n.d.	n.d.	n.d.	0.33 ± 0.01 ^{a}	2.49 ± 0.22 ^{bcdefg}	0.29 ± 0.01 ^{a}
NG20	n.d.	4.84 ± 0.34 ^{j}	n.d.	1.79 ± 0.11 ^{g}	4.87 ± 0.27 ^{b}	13.50 ± 0.85 ^{m}	n.d.
NH	n.d.	n.d.	n.d.	n.d.	n.d.	6.54 ± 0.31 ^{ijk}	n.d.
OG	n.d.	n.d.	n.d.	0.27 ± 0.02 ^{a}	n.d.	0.88 ± 0.06 ^{abcde}	n.d.
PG	n.d.	n.d.	0.79 ± 0.04 ^{a}	n.d.	n.d.	2.92 ± 0.15 ^{defg}	n.d.
PTY	n.d.	10.08 ± 0.57 ^{k}	n.d.	1.18 ± 0.10 ^{e}	n.d.	1.53 ± 0.10 ^{abcde}	n.d.
QJ	n.d.	3.78 ± 0.14 ^{i}	n.d.	2.31 ± 0.13 ^{h}	n.d.	31.68 ± 2.60 ^{o}	n.d.
QOG	n.d.	n.d.	n.d.	0.36 ± 0.02 ^{ab}	n.d.	1.78 ± 0.10 ^{abcdef}	n.d.
RN1	n.d.	0.74 ± 0.04 ^{bcde}	n.d.	0.22 ± 0.00 ^{a}	n.d.	6.58 ± 0.37 ^{ijk}	n.d.
SJY	n.d.	n.d.	n.d.	0.18 ± 0.01 ^{a}	0.51 ± 0.02 ^{a}	0.32 ± 0.01 ^{a}	n.d.
SW	n.d.	n.d.	n.d.	n.d.	n.d.	3.72 ± 0.25 ^{fgh}	n.d.
SYXX	n.d.	n.d.	n.d.	n.d.	n.d.	7.69 ± 0.44 ^{jk}	n.d.
TC	n.d.	1.00 ± 0.07 ^{def}	n.d.	n.d.	n.d.	24.34 ± 1.73 ^{n}	n.d.
TCH	n.d.	n.d.	n.d.	n.d.	n.d.	2.99 ± 0.18 ^{efg}	n.d.

WHOG	n.d.	0.84 ± 0.04 ^{cde}	n.d.	1.01 ± 0.07 ^{de}	n.d.	0.96 ± 0.06 ^{abcde}	n.d.
WZ	n.d.	1.23 ± 0.04 ^{efg}	n.d.	n.d.	n.d.	10.05 ± 0.59 ^{l}	n.d.
YHY	n.d.	n.d.	n.d.	n.d.	n.d.	0.62 ± 0.04 ^{abc}	0.50 ± 0.03 ^{b}
YL	n.d.	3.31 ± 0.18 ^{i}	n.d.	1.11 ± 0.07 ^{e}	n.d.	14.91 ± 0.74 ^{m}	n.d.
YXC	n.d.	0.34 ± 0.02 ^{abc}	n.d.	n.d.	n.d.	0.74 ± 0.04 ^{abc}	n.d.
ZXY	n.d.	n.d.	n.d.	n.d.	n.d.	0.44 ± 0.02 ^{ab}	1.24 ± 0.08 ^{c}

**Cultivars**	**Naringin**	**Hesperidin**	**Neohesperidin**	**Didymin**	**Poncirin**	**Melitidin**
AY27	3.28 ± 0.18 ^{ij}	10.20 ± 0.30 ^{fg}	0.38 ± 0.01 ^{abc}	0.26 ± 0.01 ^{ab}	0.52 ± 0.02 ^{c}	n.d.
AY30	2.96 ± 0.06 ^{hij}	17.28 ± 1.07 ^{klmn}	n.d.	0.90 ± 0.00 ^{cde}	n.d.	n.d.
AY31	n.d.	11.27 ± 0.64 ^{fg}	0.54 ± 0.04 ^{abcd}	0.40 ± 0.03 ^{abc}	n.d.	n.d.
BZH	2.07 ± 0.06 ^{fg}	16.67 ± 1.08 ^{ijklm}	3.90 ± 0.20 ^{e}	3.05 ± 0.15 ^{gh}	n.d.	n.d.
CR	1.74 ± 0.05 ^{defg}	16.75 ± 0.81 ^{jklm}	1.73 ± 0.04 ^{d}	1.26 ± 0.06 ^{de}	0.77 ± 0.05 ^{d}	n.d.
CX	n.d.	9.00 ± 0.41 ^{ef}	n.d.	1.20 ± 0.10 ^{de}	n.d.	n.d.
DF	1.27 ± 0.03 ^{bcde}	15.86 ± 1.04 ^{ijkl}	n.d.	0.76 ± 0.05 ^{bcd}	n.d.	n.d.
GAC	10.91 ± 0.51 ^{r}	3.41 ± 0.06 ^{b}	18.94 ± 0.78 ^{g}	0.51 ± 0.01 ^{abc}	0.57 ± 0.03 ^{cd}	n.d.
GN	7.02 ± 0.27 ^{q}	5.06 ± 0.16 ^{bc}	0.59 ± 0.01 ^{abcd}	n.d.	n.d.	n.d.
GOC	1.00 ± 0.07 ^{abcd}	10.60 ± 0.68 ^{fg}	1.42 ± 0.02 ^{cd}	1.20 ± 0.03 ^{de}	0.21 ± 0.01 ^{ab}	n.d.
HMR	1.55 ± 0.03 ^{cdef}	16.63 ± 1.12 ^{ijklm}	n.d.	1.14 ± 0.06 ^{de}	n.d.	n.d.
HY	4.38 ± 0.33 ^{lmn}	0.65 ± 0.03 ^{a}	4.67 ± 0.22 ^{e}	n.d.	0.06 ± 0.00 ^{a}	n.d.
KZJ22	1.14 ± 0.05 ^{abcde}	6.14 ± 0.54 ^{cd}	0.39 ± 0.02 ^{abc}	1.45 ± 0.08 ^{e}	n.d.	n.d.
MBWD	5.68 ± 0.37 ^{op}	0.22 ± 0.01 ^{a}	0.31 ± 0.02 ^{abc}	0.06 ± 0.00 ^{a}	0.06 ± 0.00 ^{a}	n.d.
MTH	0.60 ± 0.01 ^{ab}	12.18 ± 1.05 ^{gh}	0.59 ± 0.04 ^{abcd}	1.21 ± 0.03 ^{de}	n.d.	n.d.
MXG	4.42 ± 0.28 ^{mn}	0.15 ± 0.01 ^{a}	0.05 ± 0.00 ^{a}	0.05 ± 0.00 ^{a}	0.05 ± 0.00 ^{a}	0.12 ± 0.00
NG20	n.d.	17.39 ± 1.42 ^{klmn}	n.d.	2.81 ± 0.23 ^{fg}	n.d.	n.d.
NH	4.05 ± 0.28 ^{klm}	14.55 ± 0.59 ^{ij}	0.40 ± 0.02 ^{abc}	2.60 ± 0.20 ^{fg}	n.d.	n.d.
OG	3.27 ± 0.02 ^{ij}	5.98 ± 0.16 ^{cd}	22.22 ± 0.48 ^{h}	n.d.	1.96 ± 0.08 ^{e}	n.d.
PG	2.83 ± 0.10 ^{hi}	15.82 ± 0.84 ^{ijkl}	1.29 ± 0.08 ^{bcd}	0.51 ± 0.03 ^{abc}	n.d.	n.d.
PTY	3.50 ± 0.29 ^{ijk}	0.81 ± 0.03 ^{a}	4.53 ± 0.29 ^{e}	n.d.	0.06 ± 0.00 ^{a}	n.d.
QJ	3.63 ± 0.23 ^{jkl}	14.39 ± 0.77 ^{hi}	1.20 ± 0.10 ^{abcd}	6.11 ± 0.47 ^{i}	n.d.	n.d.
QOG	5.03 ± 0.29 ^{no}	10.43 ± 0.77 ^{fg}	19.54 ± 0.64 ^{g}	n.d.	4.18 ± 0.20 ^{f}	n.d.
RN1	0.42 ± 0.00 ^{a}	5.02 ± 0.21 ^{bc}	n.d.	0.95 ± 0.03 ^{cde}	n.d.	n.d.

SJY	2.84 ± 0.18 ^{hi}	0.34 ± 0.03 ^{a}	0.16 ± 0.00 ^{ab}	0.04 ± 0.00 ^{a}	n.d.	n.d.
SW	n.d.	20.04 ± 0.89 ^{o}	n.d.	2.23 ± 0.13 ^{f}	n.d.	n.d.
SYXX	n.d.	19.34 ± 1.48 ^{no}	0.43 ± 0.04 ^{abc}	2.54 ± 0.12 ^{fg}	n.d.	n.d.
TC	0.90 ± 0.06 ^{abc}	7.42 ± 0.53 ^{de}	0.96 ± 0.04 ^{abcd}	11.55 ± 0.72 ^{j}	n.d.	n.d.
TCH	0.88 ± 0.03 ^{abc}	17.73 ± 0.93 ^{lmn}	n.d.	0.72 ± 0.04 ^{bcd}	0.36 ± 0.02 ^{bc}	n.d.
WHOG	2.35 ± 0.08 ^{gh}	7.57 ± 0.25 ^{de}	16.05 ± 1.44 ^{f}	n.d.	2.09 ± 0.20 ^{e}	n.d.
WZ	0.93 ± 0.02 ^{abc}	18.26 ± 0.93 ^{mno}	n.d.	1.31 ± 0.05 ^{de}	n.d.	n.d.
YHY	6.41 ± 0.55 ^{pq}	0.16 ± 0.01 ^{a}	n.d.	n.d.	n.d.	n.d.
YL	1.83 ± 0.03 ^{efg}	15.10 ± 0.45 ^{ijk}	n.d.	3.57 ± 0.28 ^{h}	n.d.	n.d.
YXC	1.03 ± 0.05 ^{abcd}	6.44 ± 0.29 ^{cd}	0.16 ± 0.01 ^{ab}	0.72 ± 0.02 ^{bcd}	0.19 ± 0.01 ^{ab}	n.d.
ZXY	6.33 ± 0.50 ^{pq}	0.14 ± 0.00 ^{a}	n.d.	n.d.	n.d.	n.d.

n.d. —not detected. Results were the mean ± SD (n = 3) on a dried weight (g) of citrus basis. Values within each column followed by different letters were significantly different $p < 0.05$ according to Tukey's tests.

Table S2. The polymethoxylated flavonoid contents (mg/g DW) in the flavedo of the 35 citrus cultivars.

Cultivars	Monohydroxy-trimethoxyflavone (1)	Gardenin B	Monohydroxy-trimethoxyflavone (2)	trihydroxy-dimethoxyflavone	Monohydroxy-pentamethoxy flavone (1)	Isosinensetin
AY27	0.36 ± 0.01 ^{c}	n.d.	1.20 ± 0.07 ^{c}	n.d.	n.d.	1.63 ± 0.11 ^{lm}
AY30	n.d.	1.16 ± 0.07 ^{b}	2.27 ± 0.19 ^{d}	n.d.	2.78 ± 0.10 ^{e}	5.45 ± 0.23 ^{p}
AY31	n.d.	n.d.	n.d.	n.d.	n.d.	0.34 ± 0.03 ^{abcd}
BZH	0.38 ± 0.02 ^{cd}	n.d.	n.d.	n.d.	n.d.	0.87 ± 0.06 ^{fghi}
CR	n.d.	0.20 ± 0.01 ^{d}	n.d.	n.d.	n.d.	0.77 ± 0.06 ^{efgh}
CX	n.d.	n.d.	n.d.	n.d.	n.d.	0.58 ± 0.02 ^{cdefg}
DF	n.d.	n.d.	n.d.	n.d.	n.d.	n.d.
GAC	n.d.	n.d.	n.d.	n.d.	n.d.	0.90 ± 0.05 ^{ghi}
GN	0.43 ± 0.03 ^{d}	n.d.	n.d.	n.d.	0.40 ± 0.04 ^{b}	1.96 ± 0.15 ^{mn}

GOC	n.d.	n.d.	n.d.	n.d.	n.d.	0.40 ± 0.02 [abcde]
HMR	n.d.	n.d.	n.d.	3.78 ± 0.13	n.d.	n.d.
HY	n.d.	n.d.	n.d.	n.d.	n.d.	0.09 ± 0.00 [a]
KZJ	0.28 ± 0.02 [b]	n.d.	n.d.	n.d.	0.13 ± 0.01 [a]	0.99 ± 0.07 [hij]
MBWD	n.d.	n.d.	n.d.	n.d.	n.d.	0.41 ± 0.04 [abcde]
MTH	n.d.	n.d.	n.d.	n.d.	n.d.	1.28 ± 0.07 [jkl]
MXG	n.d.	n.d.	n.d.	n.d.	n.d.	0.11 ± 0.00 [a]
NG20	0.40 ± 0.03 [cd]	n.d.	n.d.	n.d.	n.d.	0.53 ± 0.01 [cdefg]
NH	n.d.	1.65 ± 0.05 [a]	n.d.	n.d.	0.51 ± 0.04 [bc]	1.45 ± 0.02 [kl]
OG	n.d.	n.d.	n.d.	n.d.	0.68 ± 0.02 [c]	2.16 ± 0.13 [n]
PG	0.64 ± 0.03 [f]	n.d.	0.52 ± 0.02 [b]	n.d.	0.68 ± 0.02 [c]	3.00 ± 0.19 [o]
PTY	n.d.	n.d.	n.d.	n.d.	n.d.	n.d.
QJ	0.58 ± 0.01 [e]	n.d.	n.d.	n.d.	n.d.	1.25 ± 0.07 [ijk]
QOG	n.d.	n.d.	n.d.	n.d.	n.d.	5.33 ± 0.48 [p]
RN1	0.13 ± 0.01 [a]	n.d.	n.d.	n.d.	n.d.	0.14 ± 0.01 [ab]
SJY	n.d.	n.d.	n.d.	n.d.	n.d.	0.14 ± 0.01 [ab]
SW	n.d.	n.d.	n.d.	n.d.	n.d.	0.28 ± 0.01 [abc]
SYXX	n.d.	n.d.	n.d.	n.d.	n.d.	0.28 ± 0.02 [abc]
TC	n.d.	0.56 ± 0.03 [c]	0.25 ± 0.01 [a]	n.d.	1.65 ± 0.12 [d]	0.51 ± 0.01 [bcdef]
TCH	n.d.	n.d.	0.25 ± 0.01 [a]	n.d.	n.d.	0.21 ± 0.01 [abc]
WHOG	n.d.	n.d.	n.d.	n.d.	n.d.	1.90 ± 0.07 [mn]
WZ	n.d.	n.d.	n.d.	n.d.	n.d.	0.32 ± 0.02 [abc]
YHY	n.d.	n.d.	n.d.	n.d.	n.d.	n.d.
YL	n.d.	n.d.	n.d.	n.d.	n.d.	0.71 ± 0.05 [defgh]
YXC	n.d.	n.d.	n.d.	n.d.	n.d.	0.27 ± 0.02 [abc]
ZXY	n.d.	n.d.	n.d.	n.d.	n.d.	n.d.

**Cultivars**	**Monohydroxy-trimethoxyflavone**	**Monohydroxy-pentamethoxyflavone(2)**	**Hexamethoxyflavone (1)**	**Sinensetin**	**Tetramethyl-*O*-isoscutellarein**	**Dihydroxy-trimethoxyflavone**
AY27	n.d.	0.27 ± 0.02 [abc]	n.d.	7.36 ± 0.41 [ij]	0.80 ± 0.03 [gh]	n.d.
AY30	n.d.	1.13 ± 0.03 [e]	n.d.	33.89 ± 2.89 [k]	n.d.	12.04 ± 0.98 [d]
AY31	n.d.	n.d.	n.d.	0.24 ± 0.01 [ab]	0.45 ± 0.01 [cdefg]	n.d.
BZH	n.d.	n.d.	n.d.	0.68 ± 0.06 [abcd]	1.37 ± 0.07 [i]	n.d.
CR	n.d.	n.d.	0.28 ± 0.01 [g]	6.33 ± 0.16 [hi]	n.d.	n.d.
CX	n.d.	n.d.	0.15 ± 0.01 [e]	0.20 ± 0.02 [a]	0.83 ± 0.07 [h]	n.d.
DF	n.d.	n.d.	n.d.	0.17 ± 0.01 [a]	0.19 ± 0.01 [abcd]	n.d.
GAC	n.d.	n.d.	n.d.	0.61 ± 0.04 [abcd]	0.37 ± 0.01 [abcdef]	n.d.
GN	n.d.	0.47 ± 0.04 [c]	n.d.	2.16 ± 0.14 [cdef]	2.90 ± 0.13 [k]	n.d.
GOC	n.d.	n.d.	n.d.	0.26 ± 0.02 [ab]	0.37 ± 0.01 [bcdef]	n.d.
HMR	n.d.	n.d.	n.d.	n.d.	n.d.	0.34 ± 0.02 [a]
HY	n.d.	n.d.	n.d.	0.07 ± 0.00 [a]	0.09 ± 0.01 [ab]	n.d.
KZJ	n.d.	0.13 ± 0.00 [a]	0.09 ± 0.01 [cd]	0.56 ± 0.04 [abcd]	0.71 ± 0.03 [fgh]	n.d.
MBWD	n.d.	n.d.	n.d.	n.d.	n.d.	n.d.
MTH	n.d.	0.29 ± 0.01 [abc]	n.d.	2.01 ± 0.06 [bcdef]	0.64 ± 0.02 [efgh]	n.d.
MXG	n.d.	n.d.	0.03 ± 0.00 [a]	n.d.	0.01 ± 0.00 [a]	n.d.
NG20	n.d.	n.d.	n.d.	0.44 ± 0.01 [abc]	0.48 ± 0.02 [defgh]	n.d.
NH	0.66 ± 0.03 [b]	n.d.	1.36 ± 0.04 [i]	8.33 ± 0.35 [j]	n.d.	0.89 ± 0.05 [ab]
OG	n.d.	2.31 ± 0.17 [f]	n.d.	2.51 ± 0.03 [ef]	2.67 ± 0.16 [j]	0.68 ± 0.04 [ab]
PG	n.d.	0.77 ± 0.02 [d]	n.d.	3.35 ± 0.05 [fg]	4.98 ± 0.28 [l]	n.d.
PTY	n.d.	n.d.	0.05 ± 0.00 [ab]	n.d.	0.10 ± 0.01 [abc]	n.d.
QJ	n.d.	n.d.	0.36 ± 0.02 [h]	0.72 ± 0.04 [abcde]	1.49 ± 0.10 [I]	n.d.
QOG	1.59 ± 0.05 [c]	4.51 ± 0.15 [g]	n.d.	4.91 ± 0.15 [gh]	5.30 ± 0.41 [l]	1.46 ± 0.11 [b]
RN1	n.d.	n.d.	0.13 ± 0.01 [de]	0.12 ± 0.00 [a]	0.13 ± 0.01 [abcd]	n.d.
SJY	n.d.	n.d.	n.d.	0.06 ± 0.00 [a]	n.d.	n.d.
SW	n.d.	n.d.	0.12 ± 0.01 [cde]	n.d.	0.21 ± 0.01 [abcd]	n.d.
SYXX	n.d.	n.d.	0.22 ± 0.01 [f]	0.20 ± 0.00 [a]	0.31 ± 0.02 [abcde]	n.d.
TC	0.17 ± 0.01 [a]	0.37 ± 0.03 [bc]	0.12 ± 0.01 [de]	3.34 ± 0.16 [fg]	n.d.	4.79 ± 0.05 [c]
TCH	n.d.	n.d.	n.d.	1.32 ± 0.06 [abcde]	n.d.	n.d.

WHOG	0.60 ± 0.02 ^{b}	2.34 ± 0.14 ^{f}	n.d.	2.32 ± 0.12 ^{def}	2.53 ± 0.13 ^{j}	0.70 ± 0.04 ^{ab}
WZ	n.d.	0.26 ± 0.01 ^{abc}	0.21 ± 0.00 ^{f}	0.45 ± 0.02 ^{abc}	0.30 ± 0.00 ^{abcde}	n.d.
YHY	n.d.	n.d.	n.d.	0.08 ± 0.01 ^{a}	n.d.	n.d.
YL	n.d.	0.18 ± 0.01 ^{ab}	0.30 ± 0.02 ^{g}	0.43 ± 0.03 ^{abc}	0.68 ± 0.04 ^{fgh}	n.d.
YXC	n.d.	0.07 ± 0.00 ^{a}	0.07 ± 0.00 ^{bc}	n.d.	0.38 ± 0.01 ^{bcdef}	n.d.
ZXY	n.d.	n.d.	n.d.	n.d.	n.d.	n.d.

**Cultivars**	**Hexamethoxyflavone (2)**	**5,7,3′,4′,5′-Pentamethoxyflavone**	**Nobiletin**	**Tetramethyl-*O*-scutellarein**	**5,4′-Dihydroxyl-3,7,8,3′-tetramethoxyflavonol**	**3,5,6,7,8,3′,4′-Heptamethoxyflavone**	**Tangeretin**
AY27	n.d.	1.22 ± 0.07	18.96 ± 0.77 ^{mn}	15.72 ± 1.01 ^{j}	0.50 ± 0.03 ^{c}	1.58 ± 0.08 ^{h}	10.63 ± 0.54 ^{h}
AY30	n.d.	n.d.	49.37 ± 3.93 ^{q}	13.54 ± 0.92 ^{i}	3.81 ± 0.12 ^{h}	1.59 ± 0.10 ^{h}	25.04 ± 1.04 ^{j}
AY31	n.d.	n.d.	5.72 ± 0.40 ^{efghij}	0.53 ± 0.02 ^{abc}	0.22 ± 0.01 ^{ab}	0.77 ± 0.03 ^{def}	2.61 ± 0.13 ^{bcdefg}
BZH	n.d.	n.d.	8.41 ± 0.19 ^{ijk}	0.26 ± 0.01 ^{abc}	n.d.	3.78 ± 0.31 ^{k}	5.10 ± 0.10 ^{g}
CR	1.37 ± 0.04 ^{f}	n.d.	10.44 ± 0.62 ^{k}	3.35 ± 0.20 ^{g}	0.29 ± 0.02 ^{b}	4.51` ± 0.14 ^{l}	2.50 ± 0.09 ^{abcdef}
CX	n.d.	n.d.	2.35 ± 0.06 ^{abcde}	0.12 ± 0 .00 ^{ab}	n.d.	0.62 ± 0.05 ^{bcde}	1.18 ± 0.09 ^{abc}
DF	n.d.	n.d.	2.90 ± 0.16 ^{abcdef}	0.27 ± 0.02 ^{abc}	n.d.	3.31 ± 0.05 ^{ij}	1.67 ± 0.11 ^{abcde}
GAC	n.d.	n.d.	6.38 ± 0.37 ^{fghij}	n.d.	n.d.	0.61 ± 0.05 ^{bcde}	2.57 ± 0.23 ^{bcdefg}
GN	n.d.	n.d.	18.09 ± 0.57 ^{m}	1.07 ± 0.06 ^{cde}	0.72 ± 0.05 ^{d}	0.38 ± 0.02 ^{abcd}	14.69 ± 0.48 ^{i}
GOC	n.d.	n.d.	3.75 ± 0.25 ^{bcdefg}	0.11 ± 0.01 ^{ab}	n.d.	1.05 ± 0.06 ^{efg}	1.65 ± 0.09 ^{abcd}
HMR	n.d.	n.d.	0.36 ± 0.03 ^{ab}	n.d.	n.d.	n.d.	n.d.
HY	n.d.	n.d.	0.87 ± 0.08 ^{abc}	0.06 ± 0.00 ^{a}	0.07 ± 0.00 ^{a}	0.30 ± 0.02 ^{abc}	0.54 ± 0.03 ^{ab}
KZJ	n.d.	n.d.	7.70 ± 0.18 ^{hijk}	0.14 ± 0.01 ^{ab}	n.d.	0.77 ± 0.06 ^{def}	3.78 ± 0.18 ^{defg}
MBWD	n.d.	n.d.	0.09 ± 0.00 ^{a}	n.d.	n.d.	n.d.	0.08 ± 0.00 ^{a}
MTH	n.d.	n.d.	21.83 ± 1.07 ^{n}	0.29 ± 0.01 ^{abc}	n.d.	2.85 ± 0.13 ^{i}	4.24 ± 0.22 ^{efg}
MXG	n.d.	n.d.	0.10 ± 0.01 ^{a}	n.d.	n.d.	0.51 ± 0.03 ^{abcd}	0.16 ± 0.01 ^{a}
NG20	n.d.	n.d.	4.36 ± 0.11 ^{cdefgh}	0.95 ± 0.02 ^{bcd}	n.d.	3.31 ± 0.06 ^{j}	1.86 ± 0.09 ^{abcdef}
NH	2.05 ± 0.12 ^{g}	n.d.	14.15 ± 0.23 ^{l}	4.79 ± 0.29 ^{h}	n.d.	5.69 ± 0.15 ^{m}	3.47 ± 0.20 ^{cdefg}

OG	n.d.	n.d.	42.20 ± 2.23 ^{p}	1.62 ± 0.08 ^{de}	1.42 ± 0.07 ^{f}	0.70 ± 0.07 ^{cdef}	33.95 ± 1.72 ^{kl}
PG	n.d.	n.d.	45.52 ± 1.21 ^{p}	1.72 ± 0.05 ^{de}	0.97 ± 0.05 ^{e}	0.47 ± 0.02 ^{abcd}	31.62 ± 2.20 ^{k}
PTY	0.11 ± 0.01 ^{ab}	n.d.	0.33 ± 0.02 ^{ab}	0.05 ± 0.00 ^{a}	n.d.	0.26 ± 0.02 ^{abc}	0.30 ± 0.00 ^{a}
QJ	0.34 ± 0.00 ^{e}	n.d.	8.78 ± 0.81 ^{jk}	0.36 ± 0.03 ^{abc}	n.d.	6.02 ± 0.20 ^{mn}	4.41 ± 0.37 ^{fg}
QOG	n.d.	n.d.	50.06 ± 3.21 ^{q}	2.79 ± 0.02 ^{g}	2.55 ± 0.03 ^{g}	0.65 ± 0.03 ^{bcdef}	43.42 ± 0.73 ^{m}
RN1	0.05 ± 0.00 ^{a}	n.d.	1.61 ± 0.07 ^{abcd}	0.05 ± 0.00 ^{a}	n.d.	1.50 ± 0.12 ^{gh}	0.68 ± 0.01 ^{ab}
SJY	n.d.	n.d.	0.23 ± 0.01 ^{ab}	0.14 ± 0.01 ^{ab}	n.d.	0.11 ± 0.00 ^{a}	0.17 ± 0.01 ^{a}
SW	0.18 ± 0.00 ^{bc}	n.d.	1.20 ± 0.04 ^{abc}	0.12 ± 0.01 ^{ab}	n.d.	1.66 ± 0.08 ^{h}	0.65 ± 0.03 ^{ab}
SYXX	0.20 ± 0.01 ^{bc}	n.d.	2.56 ± 0.12 ^{abcde}	0.19 ± 0.01 ^{ab}	n.d.	4.62 ± 0.08 ^{l}	1.23 ± 0.08 ^{abcd}
TC	0.24 ± 0.01 ^{cde}	n.d.	4.93 ± 0.16 ^{defghi}	0.68 ± 0.04 ^{abc}	1.51 ± 0.09 ^{f}	0.27 ± 0.02 ^{abc}	3.05 ± 0.21 ^{bcdefg}
TCH	0.31 ± 0.03 ^{de}	n.d.	1.61 ± 0.11 ^{abcd}	2.60 ± 0.08 ^{fg}	n.d.	1.11 ± 0.05 ^{fg}	1.10 ± 0.08 ^{abc}
WHOG	n.d.	n.d.	35.44 ± 2.16 ^{o}	1.84 ± 0.09 ^{ef}	0.73 ± 0.02 ^{d}	0.51 ± 0.01 ^{abcd}	35.00 ± 3.16 ^{l}
WZ	n.d.	n.d.	5.17 ± 0.10 ^{efghi}	0.32 ± 0.01 ^{abc}	0.60 ± 0.02 ^{cd}	6.24 ± 0.56 ^{n}	2.26 ± 0.13 ^{abcdef}
YHY	n.d.	n.d.	0.39 ± 0.02 ^{ab}	n.d.	n.d.	0.10 ± 0.00 ^{a}	n.d.
YL	0.23 ± 0.01 ^{bcd}	n.d.	5.53 ± 0.32 ^{efghij}	0.17 ± 0.00 ^{ab}	n.d.	3.41 ± 0.23 ^{jk}	2.47 ± 0.24 ^{abcdef}
YXC	n.d.	n.d.	6.89 ± 0.33 ^{ghij}	0.11 ± 0.01 ^{ab}	n.d.	0.21 ± 0.01 ^{ab}	2.38 ± 0.11 ^{abcdef}
ZXY	n.d.	n.d.	0.07 ± 0.00 ^{a}	n.d.	n.d.	n.d.	n.d.

Cultivars	6-*O*-Desmethyltangeritin/7-*O*-Desmethyltangeritin	Hexamethoxyflavone	5-Hydroxy-6,7,8,3′,4′-pentamethoxyflavone	Monohydroxy-trimethoxyflavone (3)	Monohydroxy-pentamethoxyflavone (3)	Natsudaidai	5-Hydroxy-7,8,3′,4′-tetramethoxyflavone
AY27	n.d.	n.d.	3.34 ± 0.15 ^{e}	0.83 ± 0.05 ^{c}	n.d.	0.20 ± 0.01 ^{abc}	n.d.
AY30	1.45 ± 0.13 ^{e}	n.d.	14.22 ± 0.91 ^{i}	1.12 ± 0.06 ^{d}	n.d.	n.d.	n.d.
AY31	n.d.	n.d.	0.59 ± 0.06 ^{abc}	n.d.	n.d.	n.d.	n.d.
BZH	n.d.	0.88 ± 0.05 ^{e}	1.95 ± 0.05 ^{d}	n.d.	n.d.	0.35 ± 0.01 ^{cd}	n.d.
CR	n.d.	n.d.	0.56 ± 0.02 ^{abc}	n.d.	n.d.	n.d.	n.d.
CX	n.d.	n.d.	n.d.	n.d.	n.d.	n.d.	n.d.
DF	n.d.	n.d.	0.34 ± 0.02 ^{ab}	n.d.	n.d.	0.44 ± 0.01 ^{de}	n.d.
GAC	n.d.	n.d.	0.49 ± 0.04 ^{abc}	n.d.	n.d.	n.d.	n.d.

GN	0.24 ± 0.02 ^{b}	n.d.	5.67 ± 0.24 ^{f}	0.26 ± 0.02 ^{a}	n.d.	n.d.	0.27 ± 0.00 ^{a}
GOC	n.d.	0.38 ± 0.01 ^{d}	0.47 ± 0.03 ^{abc}	n.d.	n.d.	0.15 ± 0.01 ^{ab}	n.d.
HMR	0.14 ± 0.01 ^{ab}	n.d.	n.d.	n.d.	n.d.	n.d.	n.d.
HY	n.d.	n.d.	0.12 ± 0.01 ^{a}	n.d.	n.d.	0.09 ± 0.00 ^{a}	n.d.
KZJ	n.d.	n.d.	0.50 ± 0.02 ^{abc}	n.d.	n.d.	n.d.	n.d.
MBWD	n.d.	n.d.	n.d.	n.d.	n.d.	0.09 ± 0.01 ^{a}	n.d.
MTH	n.d.	n.d.	1.29 ± 0.06 ^{cd}	n.d.	n.d.	n.d.	n.d.
MXG	n.d.	n.d.	0.08 ± 0.00 ^{a}	n.d.	n.d.	n.d.	n.d.
NG20	n.d.	n.d.	0.43 ± 0.03 ^{abc}	n.d.	0.88 ± 0.02 ^{c}	0.65 ± 0.01 ^{f}	n.d.
NH	0.46 ± 0.01 ^{c}	n.d.	1.09 ± 0.10 ^{bcd}	0.67 ± 0.05 ^{b}	n.d.	0.55 ± 0.03 ^{ef}	n.d.
OG	n.d.	n.d.	8.97 ± 0.35 ^{gh}	n.d.	n.d.	n.d.	1.14 ± 0.06 ^{b}
PG	n.d.	n.d.	9.52 ± 0.19 ^{h}	n.d.	n.d.	n.d.	n.d.
PTY	n.d.	n.d.	0.09 ± 0.00 ^{a}	n.d.	n.d.	0.08 ± 0.00 ^{a}	n.d.
QJ	1.04 ± 0.01 ^{d}	0.36 ± 0.03 ^{d}	0.69 ± 0.03 ^{abc}	n.d.	n.d.	0.33 ± 0.02 ^{cd}	n.d.
QOG	n.d.	n.d.	17.17 ± 0.97 ^{j}	n.d.	n.d.	n.d.	2.21 ± 0.13 ^{d}
RN1	0.05 ± 0.00 ^{a}	0.04 ± 0.00 ^{a}	0.10 ± 0.00 ^{a}	n.d.	0.04 ± 0.00 ^{a}	0.09 ± 0.00 ^{a}	n.d.
SJY	n.d.	n.d.	0.10 ± 0.01 ^{a}	n.d.	n.d.	n.d.	n.d.
SW	n.d.	0.12 ± 0.00 ^{b}	0.11 ± 0.01 ^{a}	n.d.	0.09 ± 0.01 ^{b}	0.19 ± 0.00 ^{abc}	n.d.
SYXX	n.d.	0.18 ± 0.01 ^{c}	0.27 ± 0.02 ^{ab}	n.d.	n.d.	1.97 ± 0.19 ^{h}	n.d.
TC	n.d.	n.d.	2.00 ± 0.12 ^{d}	n.d.	n.d.	0.28 ± 0.01 ^{bcd}	n.d.
TCH	n.d.	n.d.	n.d.	n.d.	n.d.	n.d.	n.d.
WHOG	n.d.	n.d.	8.50 ± 0.58 ^{g}	n.d.	n.d.	n.d.	1.45 ± 0.07 ^{c}
WZ	n.d.	n.d.	0.61 ± 0.01 ^{abc}	n.d.	n.d.	1.24 ± 0.10 ^{g}	n.d.
YHY	n.d.	n.d.	n.d.	n.d.	n.d.	n.d.	n.d.
YL	n.d.	n.d.	0.35 ± 0.02 ^{ab}	n.d.	n.d.	0.19 ± 0.01 ^{abc}	n.d.
YXC	n.d.	n.d.	0.45 ± 0.02 ^{abc}	n.d.	n.d.	n.d.	n.d.
ZXY	n.d.	n.d.	n.d.	n.d.	n.d.	n.d.	n.d.

n.d. —not detected. Results were the mean ± SD (n = 3) on a dried weight (g) of citrus basis. Values within each column followed by different letters were significantly different $p < 0.05$ according to Tukey's tests.

Table S3. The flavone and flavanone contents (mg/g DW) in the albedo of the 35 citrus cultivars.

Cultivars	Vicenin-2	Eriocitrin	Apigenin-8-*C*-glucoside	Neoericitrin	Diosmetin-6-*C*-glucoside	Narirutin
AY27	n.d.	n.d.	n.d.	n.d.	n.d.	2.27 ± 0.33 [a]
AY30	n.d.	n.d.	2.43 ± 0.28 [bc]	n.d.	n.d.	10.00 ± 1.45 [defghi]
AY31	n.d.	n.d.	n.d.	n.d.	n.d.	3.45 ± 0.43 [abc]
BZH	n.d.	5.46 ± 0.47 [e]	n.d.	1.66 ± 0.18 [abc]	9.05 ± 1.32 [a]	14.25 ± 2.46 [ghijk]
CR	n.d.	0.82 ± 0.09 [ab]	n.d.	n.d.	n.d.	3.99 ± 0.56 [abc]
CX	n.d.	0.25 ± 0.04 [a]	n.d.	n.d.	n.d.	5.61 ± 0.59 [abcd]
DF	n.d.	0.54 ± 0.06 [a]	n.d.	0.76 ± 0.08 [abc]	n.d.	18.43 ± 2.81 [kl]
GAC	n.d.	1.42 ± 0.08 [abc]	n.d.	10.12 ± 1.49 [e]	n.d.	1.53 ± 0.15 [a]
GN	n.d.	15.30 ± 2.25 [f]	17.32 ± 1.57 [d]	13.07 ± 0.89 [f]	n.d.	22.7 ± 1.47 [l]
GOC	n.d.	1.78 ± 0.32 [abc]	n.d.	0.72 ± 0.05 [abc]	n.d.	17.18 ± 2.74 [jkl]
HMR	n.d.	1.78 ± 0.13 [abc]	n.d.	n.d.	n.d.	8.31 ± 1.29 [bcdef]
HY	n.d.	0.23 ± 0.02 [a]	n.d.	2.05 ± 0.16 [c]	n.d.	1.54 ± 0.23 [a]
KZJ	6.84 ± 0.21	5.35 ± 0.48 [e]	3.93 ± 0.63 [c]	n.d.	n.d.	19.39 ± 0.48 [kl]
MBWD	n.d.	n.d.	n.d.	n.d.	n.d.	n.d.
MTH	n.d.	n.d.	0.65 ± 0.10 [ab]	n.d.	n.d.	0.69 ± 0.10 [a]
MXG	n.d.	n.d.	n.d.	n.d.	n.d.	n.d.
NG20	n.d.	3.86 ± 0.56 [de]	n.d.	1.57 ± 0.25 [abc]	4.65 ± 0.37 [b]	11.55 ± 0.72 [efghij]
NH	n.d.	n.d.	n.d.	n.d.	n.d.	6.13 ± 0.71 [abcde]
OG	n.d.	n.d.	n.d.	0.17 ± 0.02 [a]	n.d.	0.57 ± 0.04 [a]
PG	n.d.	n.d.	0.35 ± 0.04 [a]	n.d.	n.d.	1.72 ± 0.15 [a]
PTY	n.d.	3.14 ± 0.38 [cd]	n.d.	4.21 ± 0.55 [d]	n.d.	3.02 ± 0.18 [ab]
QJ	n.d.	2.48 ± 0.23 [bcd]	n.d.	1.81 ± 0.19 [bc]	n.d.	8.95 ± 1.01 [cdefg]
QOG	n.d.	n.d.	n.d.	0.12 ± 0.02 [a]	n.d.	0.59 ± 0.08 [a]
RN1	n.d.	2.32 ± 0.09 [bcd]	n.d.	0.86 ± 0.11 [abc]	n.d.	9.02 ± 1.31 [cdefgh]
SJY	n.d.	n.d.	n.d.	n.d.	n.d.	n.d.
SW	n.d.	n.d.	n.d.	n.d.	n.d.	15.76 ± 1.77 [ijk]
SYXX	n.d.	n.d.	n.d.	n.d.	n.d.	15.95 ± 1.06 [jk]
TC	n.d.	0.15 ± 0.02 [a]	n.d.	n.d.	n.d.	62.94 ± 7.71 [m]
TCH	n.d.	n.d.	n.d.	n.d.	n.d.	14.86 ± 1.00 [hijk]
WHOG	n.d.	0.25 ± 0.02 [a]	n.d.	0.30 ± 0.01 [ab]	n.d.	0.28 ± 0.03 [a]

WZ	n.d.	0.97 ± 0.03 ^{ab}	n.d.	n.d.	n.d.	13.61 ± 1.40 ^{fghijk}
YHY	n.d.	n.d.	n.d.	n.d.	n.d.	n.d.
YL	n.d.	3.96 ± 0.13 ^{de}	n.d.	1.48 ± 0.20 ^{abc}	n.d.	13.54 ± 0.46 ^{fghijk}
YXC	n.d.	0.42 ± 0.02 ^{a}	n.d.	n.d.	n.d.	2.78 ± 0.23 ^{ab}
ZXY	n.d.	n.d.	n.d.	n.d.	n.d.	n.d.

Cultivars	Rhoifolin	Naringin	Hesperidin	Neohesperidin	Didymin	Poncirin
AY27	n.d.	0.84 ± 0.03 ^{a}	9.71 ± 0.84 ^{cde}	0.37 ± 0.06 ^{a}	0.79 ± 0.10 ^{abcd}	0.52 ± 0.05 ^{ab}
AY30	n.d.	0.83 ± 0.14 ^{a}	15.52 ± 1.22 ^{fghi}	n.d.	5.25 ± 0.49 ^{i}	n.d.
AY31	n.d.	n.d.	10.01 ± 1.36 ^{de}	0.49 ± 0.09 ^{a}	2.91 ± 0.10 ^{gh}	n.d.
BZH	n.d.	0.75 ± 0.03 ^{a}	19.90 ± 1.53 ^{jk}	4.77 ± 0.71 ^{a}	2.91 ± 0.04 ^{gh}	n.d.
CR	n.d.	0.39 ± 0.04 ^{a}	13.00 ± 0.65 ^{efg}	1.33 ± 0.16 ^{a}	1.50 ± 0.13 ^{abcdef}	0.57 ± 0.07 ^{ab}
CX	n.d.	n.d.	17.81 ± 0.82 ^{hij}	n.d.	4.93 ± 0.45 ^{i}	n.d.
DF	n.d.	0.38 ± 0.05 ^{a}	16.44 ± 0.47 ^{ghij}	n.d.	2.60 ± 0.14 ^{fg}	n.d.
GAC	n.d.	27.36 ± 2.18 ^{ef}	1.19 ± 0.11 ^{a}	38.2 ± 6.62 ^{d}	0.18 ± 0.02 ^{a}	0.18 ± 0.02 ^{a}
GN	n.d.	9.87 ± 1.14 ^{b}	25.23 ± 1.26 ^{lm}	2.46 ± 0.14 ^{a}	1.91 ± 0.28 ^{bcdefg}	n.d.
GOC	n.d.	0.33 ± 0.05 ^{a}	14.30 ± 1.79 ^{fgh}	1.95 ± 0.17 ^{a}	2.01 ± 0.39 ^{cdefg}	0.27 ± 0.03 ^{a}
HMR	n.d.	0.40 ± 0.02 ^{a}	14.53 ± 2.12 ^{fgh}	n.d.	4.19 ± 0.79 ^{hi}	n.d.
HY	0.43 ± 0.02	21.70 ± 1.98 ^{cd}	0.70 ± 0.03 ^{a}	14.21 ± 0.64 ^{b}	n.d.	0.07 ± 0.01 ^{a}
KZJ	n.d.	1.32 ± 0.12 ^{a}	26.78 ± 1.01 ^{m}	1.77 ± 0.28 ^{a}	7.10 ± 1.08 ^{j}	n.d.
MBWD	n.d.	21.14 ± 1.75 ^{c}	n.d.	n.d.	n.d.	n.d.
MTH	n.d.	0.08 ± 0.01 ^{a}	5.65 ± 0.48 ^{bc}	0.23 ± 0.00 ^{a}	0.62 ± 0.09 ^{ab}	n.d.
MXG	n.d.	29.78 ± 3.39 ^{f}	n.d.	n.d.	n.d.	n.d.
NG20	n.d.	n.d.	16.36 ± 2.39 ^{ghij}	n.d.	1.86 ± 0.22 ^{bcdefg}	n.d.
NH	n.d.	1.07 ± 0.14 ^{a}	13.03 ± 0.39 ^{efg}	0.34 ± 0.04 ^{a}	2.23 ± 0.22 ^{efg}	n.d.
OG	n.d.	3.16 ± 0.33 ^{a}	3.66 ± 0.34 ^{ab}	52.88 ± 4.62 ^{f}	n.d.	3.65 ± 0.33 ^{d}
PG	n.d.	0.32 ± 0.05 ^{a}	6.44 ± 0.29 ^{bcd}	0.56 ± 0.07 ^{a}	0.92 ± 0.10 ^{abcde}	n.d.
PTY	n.d.	30.69 ± 3.66 ^{f}	0.89 ± 0.07 ^{a}	24.09 ± 0.90 ^{c}	n.d.	1.06 ± 0.11 ^{b}
QJ	n.d.	0.76 ± 0.06 ^{a}	11.95 ± 0.72 ^{ef}	0.82 ± 0.11 ^{a}	1.42 ± 0.25 ^{abcdef}	n.d.
QOG	n.d.	3.78 ± 0.53 ^{a}	3.58 ± 0.10 ^{ab}	46.14 ± 1.76 ^{ef}	n.d.	3.98 ± 0.45 ^{d}
RN1	n.d.	0.37 ± 0.01 ^{a}	16.81 ± 1.19 ^{ghij}	n.d.	1.66 ± 0.18 ^{bcdefg}	n.d.
SJY	n.d.	23.64 ± 3.71 ^{cde}	n.d.	n.d.	n.d.	n.d.

SW	n.d.	n.d.	16.95 ± 2.91 ^{ghij}	n.d.	2.19 ± 0.29 ^{efg}	n.d.
SYXX	n.d.	n.d.	18.97 ± 3.14 ^{ijk}	0.43 ± 0.03 ^{a}	2.62 ± 0.27 ^{fg}	n.d.
TC	n.d.	0.04 ± 0.01 ^{a}	1.20 ± 0.09 ^{a}	0.14 ± 0.03 ^{a}	17.88 ± 1.35 ^{k}	n.d.
TCH	n.d.	0.34 ± 0.02 ^{a}	22.24 ± 0.77 ^{kl}	n.d.	1.14 ± 0.11 ^{abcde}	0.49 ± 0.02 ^{ab}
WHOG	n.d.	2.31 ± 0.14 ^{a}	2.36 ± 0.10 ^{ab}	45.05 ± 5.60 ^{de}	n.d.	2.90 ± 0.38 ^{c}
WZ	n.d.	0.17 ± 0.02 ^{a}	13.40 ± 1.43 ^{efg}	n.d.	1.79 ± 0.22 ^{bcdefg}	n.d.
YHY	n.d.	26.51 ± 3.24 ^{def}	n.d.	n.d.	n.d.	n.d.
YL	n.d.	0.74 ± 0.06 ^{a}	19.97 ± 1.52 ^{jk}	n.d.	2.11 ± 0.21 ^{defg}	n.d.
YXC	n.d.	0.42 ± 0.07 ^{a}	9.30 ± 0.45 ^{cde}	0.20 ± 0.02 ^{a}	0.72 ± 0.10 ^{abc}	0.25 ± 0.04 ^{a}
ZXY	n.d.	22.19 ± 2.47 ^{cd}	n.d.	n.d.	n.d.	n.d.

n.d. —not detected. Results were the mean ± SD (n = 3) on a dried weight (g) of citrus basis. Values within each column followed by different letters were significantly different $p < 0.05$ according to Tukey's tests.

Table S4. The flavone and flavanone contents (mg/g DW) in the segment membrane of the 35 citrus cultivars.

**Cultivars**	**Vicenin-2**	**Eriocitrin**	**Apigenin-8-*C*-glucoside**	**Neoericitrin**	**Diosmetin-6-*C*-glucoside**	**Narirutin**
AY27	n.d.	n.d.	n.d.	n.d.	n.d.	1.60 ± 0.21 ^{ab}
AY30	n.d.	n.d.	1.95 ± 0.05 ^{a}	n.d.	n.d.	5.89 ± 0.38 ^{defgh}
AY31	n.d.	n.d.	n.d.	n.d.	n.d.	5.03 ± 0.36 ^{cdef}
BZH	n.d.	3.08 ± 0.30 ^{gh}	n.d.	0.94 ± 0.13 ^{bcd}	4.62 ± 0.16 ^{a}	11.74 ± 1.93 ^{jkl}
CR	n.d.	0.93 ± 0.07 ^{bc}	n.d.	n.d.	n.d.	7.17 ± 1.09 ^{efghi}
CX	n.d.	0.14 ± 0.02 ^{ab}	n.d.	n.d.	n.d.	3.88 ± 0.37 ^{bcde}
DF	n.d.	0.48 ± 0.04 ^{ab}	n.d.	0.61 ± 0.04 ^{abc}	n.d.	9.62 ± 0.58 ^{ijk}
GAC	n.d.	2.39 ± 0.30 ^{efg}	n.d.	4.98 ± 0.36 ^{g}	n.d.	1.53 ± 0.08 ^{ab}
GN	n.d.	9.36 ± 0.83 ^{j}	14.71 ± 1.99 ^{b}	7.62 ± 0.76 ^{h}	n.d.	12.38 ± 1.11 ^{kl}
GOC	n.d.	1.62 ± 0.14 ^{cde}	n.d.	0.65 ± 0.03 ^{abc}	n.d.	8.43 ± 0.61 ^{ghij}
HMR	n.d.	2.15 ± 0.29 ^{def}	n.d.	n.d.	n.d.	14.32 ± 1.93 ^{l}
HY	n.d.	1.45 ± 0.25 ^{cd}	n.d.	2.22 ± 0.40 ^{f}	n.d.	3.18 ± 0.20 ^{abcd}
KZJ	6.51 ± 0.39	3.22 ± 0.09 ^{ghi}	2.09 ± 0.36 ^{a}	n.d.	n.d.	9.44 ± 0.63^{ijk}
MBWD	n.d.	n.d.	n.d.	n.d.	n.d.	n.d.
MTH	n.d.	n.d.	1.79 ± 0.15 ^{a}	n.d.	n.d.	3.94 ± 0.20 ^{bcde}
MXG	n.d.	n.d.	n.d.	n.d.	n.d.	2.84 ± 0.38 ^{abcd}

NG20	n.d.	3.90 ± 0.36 ^{hi}	n.d.	1.63 ± 0.14 ^{def}	4.24 ± 0.60 ^{a}	10.27 ± 1.19 ^{ijk}
NH	n.d.	n.d.	n.d.	n.d.	n.d.	7.73 ± 1.05 ^{fghi}
OG	n.d.	n.d.	n.d.	0.13 ± 0.02 ^{ab}	n.d.	1.07 ± 0.21 ^{ab}
PG	n.d.	n.d.	0.42 ± 0.06 ^{a}	n.d.	n.d.	1.91 ± 0.22 ^{abc}
PTY	n.d.	0.84 ± 0.13 ^{abc}	n.d.	1.37 ± 0.16 ^{cde}	n.d.	1.82 ± 0.23 ^{abc}
QJ	n.d.	2.83 ± 0.40 ^{fg}	n.d.	1.76 ± 0.24 ^{ef}	n.d.	8.97 ± 0.75 ^{hij}
QOG	n.d.	n.d.	n.d.	0.01 ± 0.00 ^{a}	n.d.	0.13 ± 0.02 ^{a}
RN1	n.d.	2.41 ± 0.33 ^{efg}	n.d.	0.57 ± 0.06 ^{abc}	n.d.	7.98 ± 0.95 ^{fghi}
SJY	n.d.	n.d.	n.d.	n.d.	n.d.	n.d.
SW	n.d.	n.d.	n.d.	n.d.	n.d.	11.81 ± 1.40 ^{jkl}
SYXX	n.d.	n.d.	n.d.	n.d.	n.d.	13.98 ± 0.98 ^{l}
TC	n.d.	0.09 ± 0.00 ^{ab}	n.d.	n.d.	n.d.	23.21 ± 3.42 ^{m}
TCH	n.d.	n.d.	n.d.	n.d.	n.d.	5.49 ± 0.52 ^{defg}
WHOG	n.d.	0.01 ± 0.00 ^{a}	n.d.	0.02 ± 0.00 ^{a}	n.d.	0.10 ± 0.01 ^{a}
WZ	n.d.	0.51 ± 0.06 ^{ab}	n.d.	n.d.	n.d.	9.60 ± 0.55 ^{ijk}
YHY	n.d.	n.d.	n.d.	n.d.	n.d.	n.d.
YL	n.d.	4.03 ± 0.30 ^{i}	n.d.	1.26 ± 0.18 ^{cde}	n.d.	15.08 ± 1.87 ^{l}
YXC	n.d.	0.42 ± 0.01 ^{ab}	n.d.	n.d.	n.d.	1.81 ± 0.07 ^{abc}
ZXY	n.d.	n.d.	n.d.	n.d.	n.d.	n.d.

**Cultivars**	**Rhoifolin**	**Naringin**	**Hesperidin**	**Neohesperidin**	**Didymin**	**Poncirin**
AY27	n.d.	2.59 ± 0.29 ^{a}	9.30 ± 1.51 ^{cdef}	0.33 ± 0.04 ^{ab}	0.55 ± 0.05 ^{a}	0.44 ± 0.06 ^{a}
AY30	n.d.	2.15 ± 0.26 ^{a}	12.46 ± 0.63 ^{defgh}	n.d.	4.33 ± 0.31 ^{ij}	n.d.
AY31	n.d.	n.d.	10.32 ± 0.54 ^{cdefg}	0.42 ± 0.02 ^{ab}	3.11 ± 0.33 ^{efghi}	n.d.
BZH	n.d.	1.40 ± 0.18 ^{a}	12.73 ± 2.05 ^{defgh}	2.53 ± 0.39 ^{cd}	2.85 ± 0.34 ^{efg}	n.d.
CR	n.d.	1.39 ± 0.13 ^{a}	16.48 ± 0.69 ^{hi}	1.43 ± 0.10 ^{abcd}	4.11 ± 0.33 ^{hij}	0.65 ± 0.10 ^{ab}
CX	n.d.	n.d.	9.41 ± 1.58 ^{cdef}	n.d.	3.89 ± 0.20 ^{fghij}	n.d.
DF	n.d.	0.99 ± 0.05 ^{a}	12.62 ± 1.71 ^{defgh}	n.d.	2.07 ± 0.15 ^{bcde}	n.d.
GAC	n.d.	19.72 ± 1.32 ^{def}	0.91 ± 0.10 ^{a}	20.52 ± 1.47 ^{f}	n.d.	0.12 ± 0.02 ^{b}
GN	n.d.	15.75 ± 1.37 ^{cd}	15.54 ± 2.69 ^{hi}	1.53 ± 0.10 ^{abcd}	2.27 ± 0.37 ^{cde}	n.d.
GOC	n.d.	1.07 ± 0.09 ^{a}	13.28 ± 1.08 ^{efgh}	1.73 ± 0.11 ^{abcd}	4.72 ± 0.66 ^{jk}	0.24 ± 0.04 ^{ab}
HMR	n.d.	1.66 ± 0.09 ^{a}	18.67 ± 3.24 ^{i}	n.d.	2.22 ± 0.17 ^{bcde}	n.d.

HY	0.31 ± 0.06	17.69 ± 3.22 ^{de}	0.65 ± 0.06 ^{a}	8.68 ± 0.89 ^{e}	n.d.	0.05 ± 0.00 ^{ab}
KZJ	n.d.	2.60 ± 0.26 ^{a}	15.94 ± 0.94 ^{hi}	0.98 ± 0.11 ^{abcd}	4.83 ± 0.81 ^{jk}	n.d.
MBWD	n.d.	22.81 ± 1.51 ^{fg}	n.d.	n.d.	n.d.	n.d.
MTH	n.d.	0.61 ± 0.04 ^{a}	14.23 ± 2.11 ^{ghi}	0.63 ± 0.07 ^{abc}	1.54 ± 0.21 ^{abcd}	n.d.
MXG	n.d.	9.17 ± 1.26 ^{b}	n.d.	n.d.	n.d.	n.d.
NG20	n.d.	n.d.	16.63 ± 2.50 ^{hi}	n.d.	2.43 ± 0.18 ^{de}	n.d.
NH	n.d.	4.12 ± 0.33 ^{a}	15.74 ± 0.88 ^{hi}	0.38 ± 0.05 ^{ab}	4.04 ± 0.29 ^{ghij}	n.d.
OG	n.d.	3.67 ± 0.53 ^{a}	2.99 ± 0.19 ^{ab}	28.1 ± 2.23 ^{g}	n.d.	3.32 ± 0.46 ^{e}
PG	n.d.	1.36 ± 0.16 ^{a}	8.29 ± 0.08 ^{cd}	0.65 ± 0.09 ^{abc}	1.01 ± 0.10 ^{ab}	n.d.
PTY	n.d.	12.49 ± 1.96 ^{bc}	0.19 ± 0.01 ^{a}	6.96 ± 0.48 ^{e}	n.d.	0.54 ± 0.06 ^{ab}
QJ	n.d.	3.22 ± 0.33 ^{a}	12.33 ± 1.07 ^{defgh}	1.07 ± 0.12 ^{abcd}	1.07 ± 0.17 ^{abc}	n.d.
QOG	n.d.	3.13 ± 0.10 ^{a}	0.33 ± 0.02 ^{a}	2.72 ± 0.18 ^{d}	n.d.	2.59 ± 0.38 ^{d}
RN1	n.d.	1.19 ± 0.15 ^{a}	16.41 ± 1.82 ^{hi}	n.d.	2.02 ± 0.26 ^{bcde}	n.d.
SJY	n.d.	21.9 ± 3.90 ^{ef}	n.d.	n.d.	n.d.	n.d.
SW	n.d.	n.d.	13.54 ± 0.92 ^{fgh}	n.d.	2.24 ± 0.13 ^{cde}	n.d.
SYXX	n.d.	n.d.	18.51 ± 1.94 ^{i}	0.33 ± 0.02 ^{ab}	3.04 ± 0.54 ^{efgh}	n.d.
TC	n.d.	0.08 ± 0.01 ^{a}	0.70 ± 0.08 ^{a}	0.08 ± 0.01 ^{a}	6.42 ± 0.59 ^{l}	n.d.
TCH	n.d.	0.33 ± 0.06 ^{a}	6.45 ± 0.77 ^{bc}	n.d.	0.69 ± 0.07 ^{a}	0.13 ± 0.00 ^{ab}
WHOG	n.d.	2.50 ± 0.08 ^{a}	0.16 ± 0.02 ^{a}	2.14 ± 0.39 ^{bcd}	n.d.	1.90 ± 0.18 ^{c}
WZ	n.d.	0.45 ± 0.03 ^{a}	9.01 ± 1.15 ^{cde}	n.d.	5.84 ± 0.9 ^{kl}	n.d.
YHY	n.d.	26.69 ± 3.40 ^{g}	n.d.	n.d.	n.d.	n.d.
YL	n.d.	2.12 ± 0.09 ^{a}	18.07 ± 1.58 ^{i}	n.d.	2.71 ± 0.36 ^{def}	n.d.
YXC	n.d.	1.21 ± 0.09 ^{a}	9.62 ± 1.11 ^{cdef}	0.20 ± 0.03 ^{ab}	0.54 ± 0.04 ^{a}	0.27 ± 0.03 ^{ab}
ZXY	n.d.	23.46 ± 2.74 ^{fg}	n.d.	n.d.	n.d.	n.d.

n.d. —not detected. Results were the mean ± SD (n = 3) on a dried weight (g) of citrus basis. Values within each column followed by different letters were significantly different $p < 0.05$ according to Tukey's tests.

Table S5. The flavone and flavanone contents (mg/g DW) in the juice sacs of the 35 citrus cultivars.

**Cultivars**	**Vicenin-2**	**Eriocitrin**	**Apigenin-8-*C*-glucoside**	**Neoericitrin**	**Diosmetin-6-*C*-glucoside**	**Narirutin**
AY27	n.d.	n.d.	n.d.	n.d.	n.d.	0.45 ± 0.02 ^{ab}
AY30	n.d.	n.d.	0.77 ± 0.13 ^{ab}	n.d.	n.d.	1.10 ± 0.07 ^{abcdefg}

AY31	n.d.	n.d.	n.d.	n.d.	n.d.	1.45 ± 0.12 bcdefgh
BZH	n.d.	0.49 ± 0.04 de	n.d.	0.16 ± 0.01 a	0.63 ± 0.06 a	1.93 ± 0.34 fgh
CR	n.d.	0.28 ± 0.01 abcd	n.d.	n.d.	n.d.	1.55 ± 0.06 cdefgh
CX	n.d.	0.02 ± 0.00 a	n.d.	n.d.	n.d.	0.64 ± 0.06 abcd
DF	n.d.	0.18 ± 0.02 abc	n.d.	0.21 ± 0.01 ab	n.d.	2.09 ± 0.13 gh
GAC	n.d.	1.31 ± 0.14 g	n.d.	0.89 ± 0.12 bcd	n.d.	0.53 ± 0.08 abc
GN	n.d.	4.29 ± 0.24 i	5.16 ± 0.59 c	3.44 ± 0.17 f	n.d.	2.23 ± 0.28 h
GOC	n.d.	0.45 ± 0.04 cde	n.d.	0.17 ± 0.02 g	n.d.	1.88 ± 0.18 fgh
HMR	n.d.	1.64 ± 0.23 h	n.d.	n.d.	n.d.	8.73 ± 0.76 j
HY	n.d.	0.36 ± 0.01 bcd	n.d.	0.12 ± 0.01 abc	n.d.	1.67 ± 0.23 defgh
KZJ	3.34 ± 0.33	1.66 ± 0.05 h	1.11 ± 0.16 b	n.d.	n.d.	3.94 ± 0.28 i
MBWD	n.d.	n.d.	n.d.	n.d.	n.d.	n.d.
MTH	n.d.	n.d.	0.78 ± 0.08 ab	n.d.	n.d.	0.77 ± 0.10 abcde
MXG	n.d.	n.d.	n.d.	n.d.	n.d.	1.28 ± 0.15 bcdefgh
NG20	n.d.	0.15 ± 0.01 ab	n.d.	0.06 ± 0.01 ab	0.16 ± 0.01 b	0.73 ± 0.09 abcde
NH	n.d.	n.d.	n.d.	n.d.	n.d.	1.47 ± 0.13 bcdefgh
OG	n.d.	0.40 ± 0.01 bcde	n.d.	0.05 ± 0.01 ab	n.d.	0.55 ± 0.06 abc
PG	n.d.	n.d.	0.32 ± 0.04 a	n.d.	n.d.	8.44 ± 1.14 j
PTY	n.d.	0.91 ± 0.11 f	n.d.	0.35 ± 0.04 ab	n.d.	1.77 ± 0.28 efgh
QJ	n.d.	0.66 ± 0.05 ef	n.d.	0.40 ± 0.03 de	n.d.	0.74 ± 0.09 abcde
QOG	n.d.	0.26 ± 0.04 abcd	n.d.	0.01 ± 0.00 e	n.d.	0.41 ± 0.06 ab
RN1	n.d.	0.40 ± 0.06 bcde	n.d.	0.10 ± 0.01 ab	n.d.	0.98 ± 0.10 abcdef
SJY	n.d.	n.d.	n.d.	n.d.	n.d.	n.d.
SW	n.d.	n.d.	n.d.	n.d.	n.d.	1.74 ± 0.11 efgh
SYXX	n.d.	n.d.	n.d.	n.d.	n.d.	4.44 ± 0.14 i
TC	n.d.	0.16 ± 0.02 ab	n.d.	n.d.	n.d.	4.83 ± 0.79 i
TCH	n.d.	n.d.	n.d.	n.d.	n.d.	2.04 ± 0.21 fgh
WHOG	n.d.	0.28 ± 0.04 abcd	n.d.	0.09 ± 0.00 ab	n.d.	0.08 ± 0.02 a
WZ	n.d.	0.17 ± 0.02 abc	n.d.	n.d.	n.d.	1.37 ± 0.24 bcdefgh
YHY	n.d.	n.d.	n.d.	n.d.	n.d.	n.d.
YL	n.d.	0.87 ± 0.11 f	n.d.	0.33 ± 0.02 cde	n.d.	2.14 ± 0.12 gh
YXC	n.d.	0.21 ± 0.01 abcd	n.d.	n.d.	n.d.	0.62 ± 0.45 abcd

ZXY	n.d.	n.d.	n.d.	n.d.	n.d.	n.d.

**Cultivars**	**Rhoifolin**	**Naringin**	**Hesperidin**	**Neohesperidin**	**Didymin**	**Poncirin**
AY27	n.d.	1.33 ± 0.12 ^{hi}	8.02 ± 0.20 ^{j}	0.28 ± 0.05 ^{a}	0.18 ± 0.02 ^{bcde}	0.39 ± 0.07 ^{d}
AY30	n.d.	0.85 ± 0.09 ^{efg}	5.01 ± 0.74 ^{fgh}	n.d.	0.60 ± 0.06 ^{j}	n.d.
AY31	n.d.	n.d.	3.66 ± 0.24 ^{defg}	0.15 ± 0.00 ^{a}	0.54 ± 0.03 ^{ij}	n.d.
BZH	n.d.	0.23 ± 0.02 ^{abc}	1.86 ± 0.25 ^{abcd}	0.38 ± 0.04 ^{a}	0.30 ± 0.02 ^{efg}	n.d.
CR	n.d.	0.47 ± 0.07 ^{abcde}	5.17 ± 0.96 ^{fgh}	0.51 ± 0.04 ^{a}	0.39 ± 0.07 ^{ghi}	0.23 ± 0.03 ^{c}
CX	n.d.	n.d.	0.98 ± 0.05 ^{ab}	n.d.	0.27 ± 0.01 ^{defg}	n.d.
DF	n.d.	0.36 ± 0.02 ^{abcd}	5.43 ± 0.42 ^{gh}	n.d.	0.31 ± 0.04 ^{efg}	n.d.
GAC	n.d.	2.77 ± 0.25 ^{k}	n.d.	3.68 ± 0.24 ^{c}	n.d.	n.d.
GN	n.d.	1.87 ± 0.02 ^{j}	4.47 ± 0.50 ^{efgh}	0.52 ± 0.09 ^{a}	0.07 ± 0.01 ^{ab}	n.d.
GOC	n.d.	0.28 ± 0.02 ^{abc}	3.38 ± 0.26 ^{def}	0.39 ± 0.05 ^{a}	0.17 ± 0.02 ^{abcde}	0.06 ± 0.01 ^{ab}
HMR	n.d.	1.37 ± 0.22 ^{hi}	15.4 ± 1.97 ^{k}	n.d.	4.22 ± 0.07 ^{n}	n.d.
HY	0.02 ± 0.00	2.42 ± 0.28 ^{k}	0.04 ± 0.00 ^{a}	0.92 ± 0.11 ^{ab}	n.d.	0.01 ± 0.00 ^{a}
KZJ	n.d.	1.34 ± 0.06 ^{hi}	7.95 ± 0.24 ^{j}	0.45 ± 0.05 ^{a}	1.47 ± 0.12 ^{m}	n.d.
MBWD	n.d.	0.66 ± 0.10 ^{cdef}	n.d.	n.d.	n.d.	n.d.
MTH	n.d.	0.30 ± 0.00 ^{abc}	5.95 ± 0.64 ^{hi}	0.31 ± 0.01 ^{a}	0.37 ± 0.04 ^{fgh}	n.d.
MXG	n.d.	3.43 ± 0.44 ^{l}	n.d.	n.d.	n.d.	n.d.
NG20	n.d.	n.d.	0.62 ± 0.05 ^{a}	n.d.	0.51 ± 0.06 ^{hij}	n.d.
NH	n.d.	1.55 ± 0.24 ^{ij}	7.37 ± 0.97 ^{ij}	0.17 ± 0.02 ^{a}	0.40 ± 0.05 ^{ghi}	n.d.
OG	n.d.	0.76 ± 0.09 ^{defg}	1.42 ± 0.22 ^{abc}	11.64 ± 1.92 ^{e}	n.d.	0.61 ± 0.06 ^{e}
PG	n.d.	1.17 ± 0.17 ^{ghi}	7.62 ± 1.09 ^{ij}	0.63 ± 0.06 ^{a}	0.78 ± 0.14 ^{k}	n.d.
PTY	n.d.	2.38 ± 0.23 ^{k}	0.13 ± 0.02 ^{a}	2.44 ± 0.22 ^{bc}	n.d.	0.01 ± 0.00 ^{a}
QJ	n.d.	0.63 ± 0.08 ^{bcdef}	2.77 ± 0.53 ^{bcde}	0.24 ± 0.03 ^{a}	0.01 ± 0.00 ^{a}	n.d.
QOG	n.d.	0.63 ± 0.10 ^{bcdef}	0.32 ± 0.02 ^{a}	7.40 ± 1.00 ^{d}	n.d.	0.49 ± 0.03 ^{d}
RN1	n.d.	0.19 ± 0.03 ^{ab}	2.86 ± 0.26 ^{cde}	n.d.	0.05 ± 0.01 ^{ab}	n.d.
SJY	n.d.	0.94 ± 0.06 ^{fgh}	n.d.	n.d.	n.d.	n.d.
SW	n.d.	n.d.	5.07 ± 0.34 ^{fgh}	n.d.	0.16 ± 0.01 ^{abcde}	n.d.
SYXX	n.d.	n.d.	3.51 ± 0.33 ^{def}	0.07 ± 0.01 ^{a}	0.61 ± 0.07 ^{j}	n.d.
TC	n.d.	0.15 ± 0.01 ^{a}	1.30 ± 0.11 ^{abc}	0.14 ± 0.01 ^{a}	1.00 ± 0.01 ^{l}	n.d.
TCH	n.d.	0.14 ± 0.01 ^{a}	3.08 ± 0.43 ^{cde}	n.d.	0.10 ± 0.01 ^{abc}	0.05 ± 0.01 ^{ab}

WHOG	n.d.	0.78 ± 0.01 ^{defg}	0.66 ± 0.06 ^{a}	7.64 ± 0.45 ^{d}	n.d.	0.47 ± 0.08 ^{d}
WZ	n.d.	0.13 ± 0.01 ^{a}	3.13 ± 0.32 ^{cde}	n.d.	0.12 ± 0.01 ^{abcd}	n.d.
YHY	n.d.	0.78 ± 0.03 ^{defg}	n.d.	n.d.	n.d.	n.d.
YL	n.d.	0.46 ± 0.01 ^{abcde}	4.55 ± 0.57 ^{efgh}	n.d.	0.21 ± 0.02 ^{bcdef}	n.d.
YXC	n.d.	0.81 ± 0.03 ^{defg}	5.44 ± 0.23 ^{gh}	0.18 ± 0.01 ^{a}	0.26 ± 0.01 ^{cdefg}	0.14 ± 0.01 ^{bc}
ZXY	n.d.	1.12 ± 0.06 ^{ghi}	n.d.	n.d.	n.d.	n.d.

n.d. —not detected. Results were the mean ± SD (n = 3) on a dried weight (g) of citrus basis. Values within each column followed by different letters were significantly different $p < 0.05$ according to Tukey's tests.

Table S6. Structure of flavonoid compounds identified in 35 citrus varieties

Peak no.	Tentative Compounds	Structure	Remarks
Flavone C-glycosides			
1	Vicenin-2	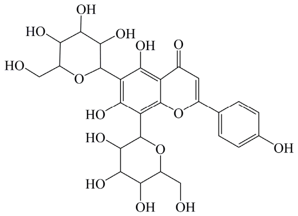	
3	Apigenin-8-*C*-glucoside	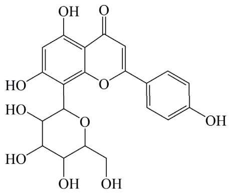	
5	Diosmetin-6-*C*-glucoside	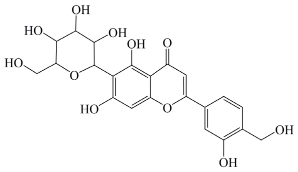	
Flavanone O-glycosides			

2	Eriocitrin	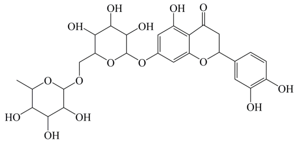
4	Neoeriocitrin	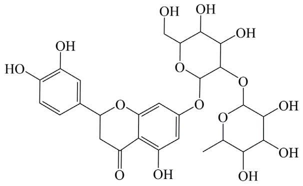
6	Narirutin	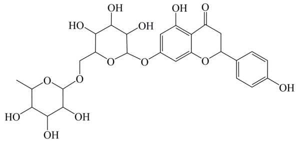
8	Naringin	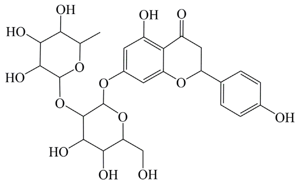
9	Hesperidin	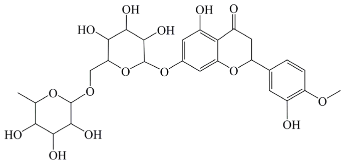

10	Neohesperidin	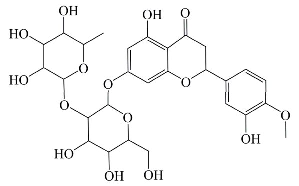
11	Didymin	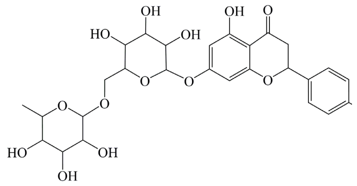
12	Poncirin	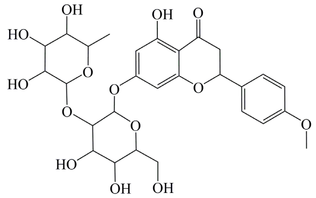
15	Melitidin	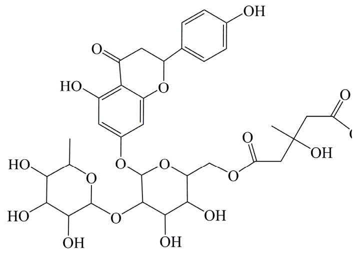

Flavone O-glycoside

7	Rhoifolin	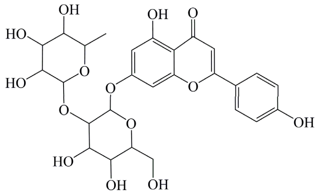	
Polymethoxyflavonoids			
13	Monohydroxy-trimethoxyflavone (1)	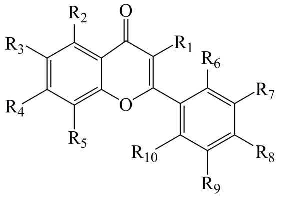	R_{X1} : OH<br>R_{X2}, R_{X3}, R_{X4} : CH_3
14	Gardenin B	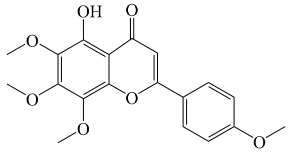	
16	Monohydroxy-trimethoxyflavone (2)	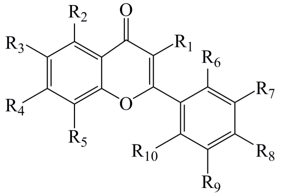	R_{X1} : OH<br>R_{X2}, R_{X3}, R_{X4} : CH_3
17	Trihydroxy-dimethoxyflavone	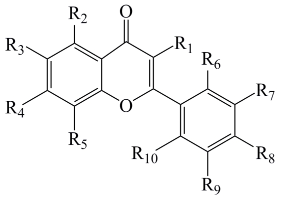	R_{X1}, R_{X2}, R_{X3} : OH<br>R_{X4}, R_{X5} : CH_3

18	Monohydroxy-pentamethoxyflavone (1)	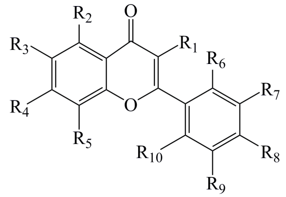	R_{X1} : OH<br>R_{X2}, R_{X3}, R_{X4}, R_{X5}, R_{X6} : CH_3
19	Isosinensetin	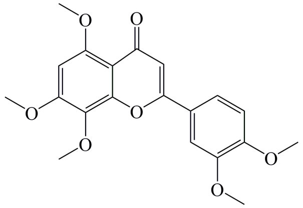	
20	Monohydroxy-tetramethoxyflavone	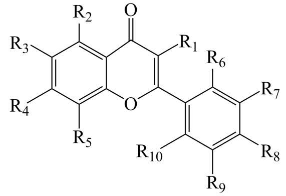	R_{X1} : OH<br>R_{X2}, R_{X3}, R_{X4}, R_{X5} : CH_3
21	Monohydroxy-pentamethoxyflavone (2)	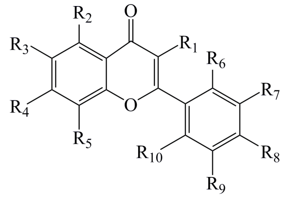	R_{X1} : OH<br>R_{X2}, R_{X3}, R_{X4}, R_{X5}, R_{X6} : CH_3
22	Hexamethoxyflavone (1)	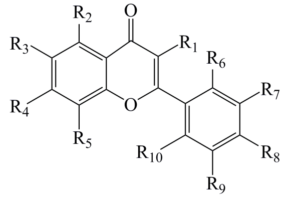	R_{X1}, R_{X2}, R_{X3}, R_{X4}, R_{X5}, R_{X6} : CH_3

23	Sinensetin	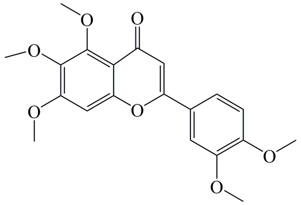	
24	Tetramethyl-*O*-isoscutellarein	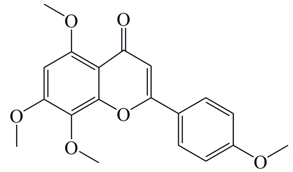	
25	Dihydroxy-trimethoxyflavone	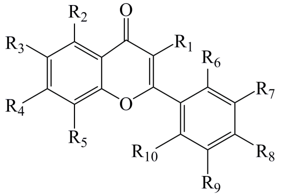	R_{X1}, R_{X2} : OH<br>R_{X3}, R_{X4}, R_{X5} : CH_3
26	Hexa-*O*-methylgossypetin	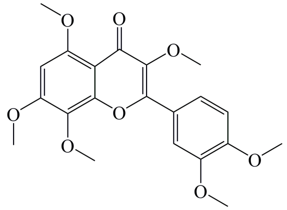	
27	5,7,3′,4′,5′-Pentamethoxyflavone	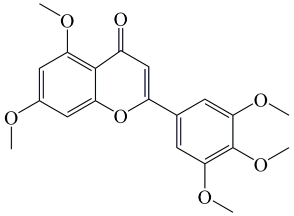	

28	Nobiletin	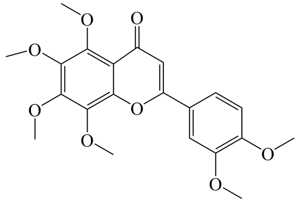
29	Tetramethyl-*O*-scutellarein	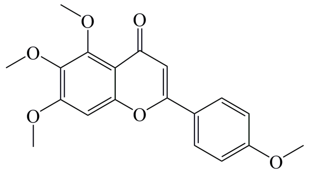
30	5,4′-Dihydroxyl-3,7,8,3′-tetramethoxyflavonol	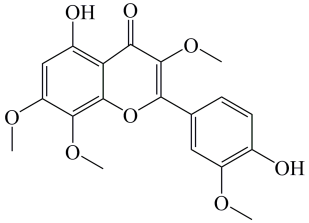
31	3,5,6,7,8,3',4'-Heptamethoxyflavone	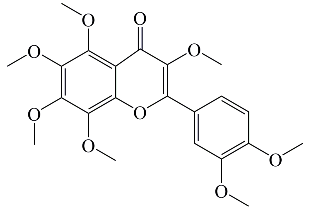
32	Tangeretin	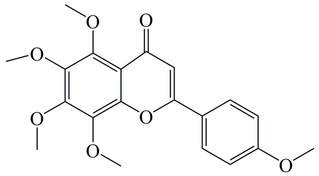

33	6-*O*-desmethyltangeritin	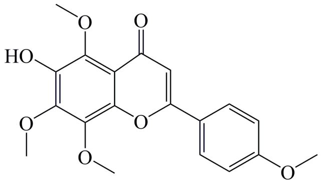	
	7-*O*-desmethyltangeritin	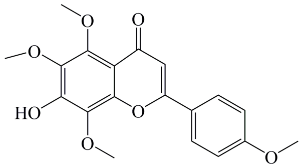	
34	Hexamethoxyflavone (2)	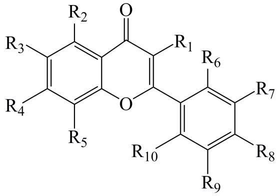	R_{X1}, R_{X2}, R_{X3}, R_{X4}, R_{X5}, R_{X6} : CH_3
35	5-Hydroxy-6,7,8,3′,4′-pentamethoxyflavone	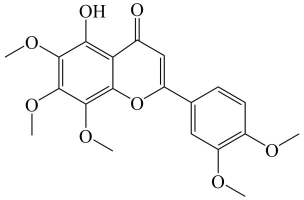	
36	Monohydroxy-trimethoxyflavone	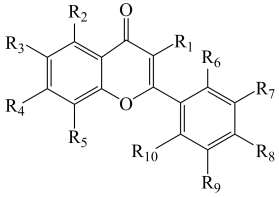	R_{X1} : OH<br>R_{X2}, R_{X3}, R_{X4} : CH_3

37	Monohydroxy-pentamethoxyflavone (3)	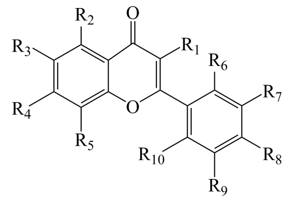	R_{X1} : OH<br>R_{X2}, R_{X3}, R_{X4}, R_{X5}, R_{X6} : CH_3
38	Natsudaidai	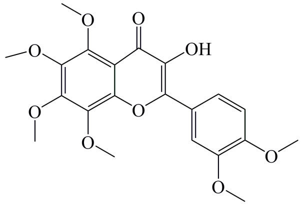	
39	5-Hydroxy-7,8,3′,4′-tetramethoxyflavone	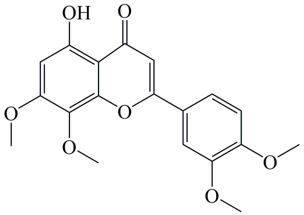	

Table S7. Pearson's correlation coefficients among bioactive traits and individual flavonoid compounds in flavedo

Flavonoids	Total Phenolic	DPPH	FRAP	ORAC	CUPRAC	APC Index	1/IC_{50} SGC-7901	1/IC_{50} BGC-823	1/IC_{50} AGS
Vicenin-2	0.155	0.093	0.126	0.339 *	0.141	0.188	0.086	0.071	0.092
Eriocitrin	0.150	0.306	0.031	0.122	0.151	0.170	−0.049	−0.101	−0.082
Apigenin-8-*C*-glucoside	0.227	0.122	0.119	0.145	0.205	0.161	0.280	0.207	0.183
Neoeriocitrin	0.012	0.122	−0.116	0.015	0.057	0.018	0.135	0.019	0.043

Diosmetin-6-*C*-glucoside	0.091	0.155	0.082	0.062	0.072	0.106	−0.041	−0.035	−0.044
Narirutin	0.451 **	0.449 **	0.310	0.490 **	0.484 **	0.477 **	0.320	0.230	0.354 *
Rhoifolin	−0.442 **	−0.310	−0.320	−0.290	−0.497 **	−0.396 *	−0.378 *	−0.366 *	−0.361 *
Naringin	−0.220	0.026	−0.040	−0.280	−0.280	−0.140	0.032	−0.020	−0.030
Hesperidin	0.546 **	0.320	0.388 *	0.349 *	0.558 **	0.467 **	0.320	0.373 *	0.371 *
Neohesperidin	0.053	0.030	0.066	0.094	0.086	0.075	0.241	0.153	0.172
Didymin	0.502 **	0.474 **	0.399 *	0.570 **	0.485 **	0.558 **	0.289	0.239	0.353 *
Poncirin	0.020	−0.140	−0.050	0.130	0.090	0.000	0.312	0.302	0.309
Monohydroxy-trimethoxyflavone (1)	0.197	0.120	0.107	0.105	0.199	0.145	0.322	0.246	0.330
Gardenin B	0.462 **	0.445 **	0.498 **	0.280	0.452 **	0.477 **	0.481 **	0.476 **	0.529 **
Melitidin	−0.140	0.004	0.016	−0.377 *	−0.290	−0.150	−0.110	−0.100	−0.130
Monohydroxy-trimethoxyflavone (2)	0.384 *	0.290	0.464 **	0.200	0.300	0.363 *	0.336 *	0.320	0.321
Trihydroxy-dimethoxyflavone	0.301	0.352 *	0.331	0.117	0.320	0.321	−0.120	−0.130	−0.080
Monohydroxy-pentamethoxyflavone (1)	0.609 **	0.419 *	0.332	0.534 **	0.476 **	0.524 **	0.362 *	0.310	0.362 *
Isosinensetin	0.435 **	0.210	0.381 *	0.320	0.457 **	0.380 *	0.580 **	0.522 **	0.533 **
Monohydroxy-tetramethoxyflavone	0.094	−0.030	0.007	0.197	0.162	0.080	0.351 *	0.335 *	0.415 *
Monohydroxy-pentamethoxyflavone (2)	0.160	−0.010	0.094	0.267	0.215	0.145	0.341 *	0.309	0.343 *
Hexamethoxyflavone (1)	0.170	0.201	0.139	0.056	0.260	0.183	0.405 *	0.419 *	0.459 **
Sinensetin	0.513 **	0.425 *	0.584 **	0.285	0.463 **	0.501 **	0.467 **	0.458 **	0.449 **

Tetramethyl-*O*-isoscutellarein	0.077	−0.030	0.034	0.156	0.142	0.073	0.356 *	0.269	0.300
Dihydroxy-trimethoxyflavone	0.576 **	0.473 **	0.322	0.484 **	0.476 **	0.482 **	0.310	0.260	0.310
Hexa-*O*-methylgossypetin	0.192	0.247	0.179	0.062	0.283	0.217	0.451 **	0.529 **	0.494 **
5,7,3′,4′,5′-Pentamethoxyflavone	−0.130	−0.070	0.001	−0.170	−0.180	−0.110	0.211	0.298	0.273
Nobiletin	0.378 *	0.200	0.373 *	0.300	0.400 *	0.354 *	0.587 **	0.530 **	0.534 **
Tetramethyl-*O*-scutellarein	0.256	0.220	0.372 *	0.075	0.212	0.257	0.483 **	0.537 **	0.507 **
5,4′-Dihydroxyl-3,7,8,3′-tetramethoxyflavonol	0.470 **	0.333	0.515 **	0.457 **	0.438 **	0.490 **	0.386 *	0.349 *	0.375 *
3,5,6,7,8,3′,4′-Heptamethoxyflavone	0.280	0.226	0.184	0.090	0.371 *	0.240	0.362 *	0.397 *	0.395 *
Tangeretin	0.237	0.086	0.220	0.267	0.277	0.231	0.478 **	0.408 *	0.417 *
6-*O*-desmethyltangeritin/7-*O*-desmethyltangeritin	0.526 **	0.487 **	0.520 **	0.220	0.494 **	0.493 **	0.495 **	0.362 *	0.450 **
Hexamethoxyflavone (2)	0.120	0.171	0.030	0.006	0.163	0.103	0.223	0.138	0.152
5-Hydroxy-6,7,8,3′,4′-pentamethoxyflavone	0.335 *	0.180	0.344 *	0.310	0.339 *	0.320	0.454 **	0.391 *	0.413 *
Monohydroxy-trimethoxyflavone	0.328	0.342 *	0.478 **	0.051	0.259	0.334	0.444 **	0.449 **	0.467 **
Monohydroxy-pentamethoxyflavone (3)	0.082	0.021	0.000	0.168	0.001	0.047	−0.170	−0.140	−0.140

Natsudaidai	0.062	0.023	0.009	0.052	0.105	0.048	−0.060	−0.040	−0.020
5-Hydroxy-7,8,3′,4′-tetramethoxyflavone	0.022	−0.080	−0.030	0.168	0.086	0.026	0.272	0.238	0.257

1/IC_{50} means the reciprocal value of IC_{50}; One and two asterisks represent statistical significance at $p < 0.05$ and $p < 0.01$, respectively

Table S8. Pearson's correlation coefficients among bioactive traits and individual flavonoid compounds in albedo

Flavonoids	Total Phenolic	DPPH	FRAP	ORAC	CUPRAC	APC Index
Vicenin-2	0.028	−0.066	0.053	−0.046	−0.033	−0.032
Eriocitrin	0.050	−0.030	−0.058	0.175	0.051	0.050
Apigenin-8-*C*-glucoside	0.035	−0.001	−0.044	0.156	0.049	0.056
Neoeriocitrin	0.433 **	0.259	0.229	0.321	0.220	0.337 *
Diosmetin-6-*C*-glucoside	−0.071	0.026	0.021	−0.127	0.055	−0.011
Narirutin	−0.157	−0.140	−0.186	0.517 **	−0.089	0.048
Rhoifolin	0.088	0.016	−0.134	0.169	0.005	0.025
Naringin	0.406 *	0.072	−0.013	0.094	−0.227	−0.021
Hesperidin	−0.414 *	−0.239	−0.163	−0.083	−0.047	−0.172
Neohesperidin	0.683 **	0.280	0.301	0.432 **	0.718 **	0.565 **
Didymin	−0.118	−0.015	−0.038	0.367 *	−0.051	0.094
Poncirin	0.407 *	0.056	0.085	0.309	0.618 **	0.351 *

One and two asterisks represent statistical significance at $p < 0.05$ and $p < 0.01$, respectively

Table S9. Pearson's correlation coefficients among bioactive traits and individual flavonoid compounds in segment membrane

Flavonoids	Total Phenolic	DPPH	FRAP	ORAC	CUPRAC	APC Index
Vicenin-2	−0.048	−0.266	−0.120	−0.099	−0.218	−0.234
Eriocitrin	0.303	−0.130	−0.035	0.230	−0.087	0.014
Apigenin-8-*C*-glucoside	0.075	−0.123	−0.104	0.110	−0.167	−0.082
Neoeriocitrin	0.450 **	−0.049	0.113	0.344 *	0.097	0.195
Diosmetin-6-*C*-glucoside	−0.004	−0.028	0.017	0.065	−0.053	0.006
Narirutin	0.184	0.199	0.221	0.194	0.039	0.225
Rhoifolin	0.140	0.017	0.051	0.195	0.324	0.211
Naringin	0.370 *	−0.094	−0.077	0.592 **	0.468 **	0.346 *
Hesperidin	−0.066	−0.002	−0.014	−0.223	−0.243	−0.178
Neohesperidin	0.555 **	0.143	0.169	0.247	0.014	0.204
Didymin	−0.093	0.070	0.075	−0.037	−0.147	−0.019
Poncirin	0.199	0.018	−0.156	0.004	−0.327	−0.153

One and two asterisks represent statistical significance at $p < 0.05$ and $p < 0.01$, respectively

Table S10. Pearson's correlation coefficients among bioactive traits and individual flavonoid compounds in juice sacs

Flavonoids	Total Phenolic	DPPH	FRAP	ORAC	CUPRAC	APC Index
Vicenin-2	0.078	−0.381 *	−0.203	0.433 **	−0.338 *	−0.147
Eriocitrin	0.061	−0.114	−0.128	0.105	−0.041	−0.057
Apigenin-8-*C*-glucoside	0.012	−0.068	−0.128	−0.043	−0.025	−0.096
Neoeriocitrin	0.021	0.035	−0.038	−0.055	0.05	−0.005

Diosmetin-6-*C*-glucoside	−0.044	0.119	0.129	−0.248	0.317	0.091
Narirutin	0.215	−0.02	0.001	0.076	0.013	0.029
Rhoifolin	0.155	0.286	0.192	−0.132	0.412 *	0.261
Naringin	0.247	0.138	0.262	0.308	0.183	0.336 *
Hesperidin	0.188	−0.142	0.007	0.182	−0.098	−0.009
Neohesperidin	0.377 *	−0.055	−0.11	0.309	−0.097	0.042
Didymin	0.049	−0.13	−0.031	0.004	−0.042	−0.074
Poncirin	0.383 *	−0.122	−0.068	0.347 *	−0.093	0.047

One and two asterisks represent statistical significance at $p < 0.05$ and $p < 0.01$, respectively
